# ﻿Exceptional larval morphology of nine species of the *Anastrephamucronota* species group (Diptera, Tephritidae)

**DOI:** 10.3897/zookeys.1127.84628

**Published:** 2022-11-03

**Authors:** Erick J. Rodriguez, Gary J. Steck, Matthew R. Moore, Allen L. Norrbom, Jessica Diaz, Louis A. Somma, Raul Ruiz-Arce, Bruce D. Sutton, Norma Nolazco, Alies Muller, Marc A. Branham

**Affiliations:** 1 Department of Entomology and Nematology, University of Florida, Gainesville, FL, USA University of Florida Gainesville United States of America; 2 Florida Department of Agriculture and Consumer Services, Division of Plant Industry (FDACS/DPI), Gainesville, FL, USA Florida Department of Agriculture and Consumer Services, Division of Plant Industry Gainesville United States of America; 3 Systematic Entomology Laboratory, USDA, ARS, c/o Smithsonian Institution, Washington, DC, USA c/o Smithsonian Institution Washington United States of America; 4 USDA APHIS PPQ S and T Insect Management and Molecular Diagnostic Laboratory, 22675 N. Moorefield Road, Edinburg, TX 78541, USA USDA APHIS PPQ S and T Insect Management and Molecular Diagnostic Laboratory Edinburg United States of America; 5 Research Associate, Department of Entomology, Smithsonian Institution, USNM, Gainesville, FL, USA Smithsonian Institution Gainesville United States of America; 6 Centro de Diagnostico de Sanidad Vegetal, Servicio Nacional de Sanidad Agraria, Av. La Molina 1915, La Molina, Peru Centro de Diagnostico de Sanidad Vegetal, Servicio Nacional de Sanidad Agraria La Molina Peru; 7 (retired) Ministry of Agriculture, Animal Husbandry and Fisheries, Paramaribo, Suriname Ministry of Agriculture Paramaribo Suriname

**Keywords:** Biology, distribution, fruit fly, host plant, larvae, taxonomy

## Abstract

*Anastrepha* is the most diverse and economically important genus of Tephritidae in the American tropics and subtropics. The striking morphology of the third instars of *Anastrephacaballeroi* Norrbom, *Anastrephacrebra* Stone, *Anastrephahaplacantha* Norrbom & Korytkowski, *Anastrephakorytkowskii* Norrbom, *Anastrephanolazcoae* Norrbom & Korytkowski, and three newly discovered and as yet formally unnamed species (*Anastrepha* sp. Peru-82, Anastrephasp.nr.protuberans, and *Anastrepha* sp. Sur-16), and the more typical morphology of *Anastrephaaphelocentema* Stone, are described using light and scanning electron microscopy. To contribute to a better understanding of the interspecific and intraspecific variation among species in the *mucronota* species group and facilitate phylogenetic studies, we integrate molecular and morphological techniques to confirm the identity and describe third instars. Larva-adult associations and the identification of described larvae were confirmed using DNA barcodes. We provide diagnostic characters to distinguish larvae among these nine species of the *mucronota* group and separate them from those of the 29 other *Anastrepha* species previously described. We introduce the vertical comb-like processes on the oral margin as a novel character, and the unusual character states, including position and shape of the preoral lobe, and dentate or fringed posterior margins of the oral ridges and accessory plates. Our comparative morphology concurs with most previously inferred phylogenetic relationships within the *mucronota* group.

## ﻿Introduction

*Anastrepha* Schiner is the most species-rich and economically important genus of fruit flies in the American tropics and subtropics, comprising 328 described species to date (Norrbom et al. 2012, 2015, 2018, 2021; Rodríguez Clavijo and Norrbom 2021), for which third instars of only 29 species (~ 9%) have been described (Rodriguez et al. 2021). Some *Anastrepha* species are well known major economic pests, including *Anastrephafraterculus* (Wiedemann) sensu lato (South American fruit fly complex), *Anastrephaludens* (Loew) (Mexican fruit fly), *Anastrephaobliqua* (Macquart) (West Indian fruit fly), *Anastrephaserpentina* (Wiedemann) (sapote fruit fly), *Anastrephastriata* Schiner (guava fruit fly), and *Anastrephasuspensa* (Loew) (Caribbean fruit fly). Approximately 40 other species are considered minor pests, most with more restricted ranges of edible host plants (Norrbom et al. 1999; Norrbom 2004; Steck et al. 2019). Despite the economic significance and high diversity of *Anastrepha*, there is limited information on the taxonomy of the immature stages.

*Anastrepha* is currently divided into 27 species groups (Norrbom et al. 1999; Mengual et al. 2017; Norrbom et al. 2018; Steck et al. 2019), of which the *mucronota* group (52 species) is the most diverse (Norrbom et al. 2012; Steck et al. 2019), although it may not be monophyletic (Mengual et al. 2017). The *mucronota* group contains several minor pest species, including *A.atrox* (Aldrich), *A.bezzii* Lima, *A.mucronota* Stone, and *A.nolazcoae* Norrbom & Korytkowski (Steyskal 1977; Norrbom and Kim 1988; White and Elson Harris 1992; Norrbom and Korytkowski 2011; Norrbom et al. 2015; Steck et al. 2019). Of these four species, a description of the larva has been published only for *A.mucronota* (as *A.nunezae*, Steyskal 1977), but it is brief and lacks measurement data and details of major morphological structures. *Anastrephamucronota* has been reared from *Annonacherimola* Mill. (cherimoya) (Molineros et al. 1992), and *Quararibeacordata* (Bonpl.) Vischer (yellow zapote), and the latter plant is also a host of *A.nolazcoae* (Steyskal 1977; Yepes and Vélez 1989; Carrejo and González 1994, 1999; Norrbom and Korytkowski 2011). *Anastrephaatrox* has been reared from *Annonacherimola* and *Pouterialucuma* (Ruiz and Pav.) Kuntze (lucumo, lucma, lucuma in Spanish) (Molineros et al. 1992; Peña and Bennett 1995; Tigrero 1998, 2009; Korytkowski 2001; Norrbom 2004). *Anastrephabezzii* has been reported to attack fruits of *Sterculiaapetala* (Jacq.) H. Karst. (Panama tree, camoruro, anacaguita) and *Sterculiacuriosa* (Vell.) Taroda (Norrbom and Kim 1988; Norrbom 1991, 2004; White and Elson-Harris 1992; Hernández-Ortiz 2007; Zucchi and Moraes 2008). Of those five hosts, *Q.cordata*, *P.lucuma*, and *A.cherimola* have edible fruit. Pulp of *Q.cordata* is eaten fresh as dessert because of its sweet flavor, and it is commonly grown on small farms and has the potential to become a more important commercial crop in native areas (Martin et al. 1987).

The host plant relationships of *Anastrepha* are poorly known and are reported for only 127 (39%) *Anastrepha* species, including 15 species in the *mucronota* group (Norrbom 2004; CoFFHI 2020). Host plant and geographic distribution data are important to regulate international trade, prevent introduction of invasive pests, and facilitate their control or eradication. Host plant information for both pest and non-pest *Anastrepha* species will also likely contribute to understanding the evolution of *Anastrepha* (Aluja 1994; Norrbom et al. 1999; Aluja and Mangan 2008; Mansell 2017). New host plant and distribution records for six species within the *mucronota* group are reported here.

Molecular data sets produced for the study of phylogenetic relationships within *Anastrepha*, as well as the development of identification tools, have included species in the *mucronota* group. Cytochrome oxidase c subunit I (COI) barcodes are available for 22 species (42%) of the 52 described species within the *mucronota* group (Barr et al. 2017; Mengual et al. 2017), including six species for which larvae are described in this study: *A.aphelocentema* Stone, *A.caballeroi* Norrbom, *A.crebra* Stone, *A.haplacantha* Norrbom & Korytkowski, *A.korytkowskii* Norrbom, and *A.nolazcoae*. Previously published and new COI sequence data from reared adult flies in this study proved valuable for confirming the identity of these larvae and thereby contributing to their accurate description. This work will contribute to a better understanding of the morphological variation among species in the *mucronota* group, facilitate future phylogenetic studies, and improve capabilities for the accurate identification of larvae.

The scope of this study is to describe and illustrate the third instars of *A.aphelocentema*, *A.caballeroi*, *A.crebra*, *A.haplacantha*, *A.korytkowskii*, *A.nolazcoae*, and three as yet unnamed species here identified by code names, including *Anastrepha* sp. Peru-82, Anastrephasp.nr.protuberans, and *Anastrepha* sp. Sur-16, collected from naturally-infested fruits in Mexico, Ecuador, Peru, and Suriname. We provide diagnostic morphological characters that are useful for distinguishing larvae of these nine species of the *mucronota* group. These characters are effective for separating larvae of *A.nolazcoae* from all other *Anastrepha* species that also feed on yellow zapote (*Quararibeacordata*), including those in the *mucronota* group (*A.mucronota*), the *fraterculus* group (*A.fraterculus*, s. l.), and the *striata* group (*A.striata*). Finally, we discuss relationships within the *mucronota* species group based on a novel character and several unusual character states in the larvae including the position of the preoral organ and shape of the preoral lobe, the dentate or fringed posterior margins of the oral ridges and accessory plates, and the vertical comb-like processes on the oral margin.

## ﻿Materials and methods

### ﻿Collecting, rearing, and preservation

For Peruvian, Ecuadorian, and Surinamese samples, fallen fruits were collected and transported to a screened rearing room in 1- or 2-liter plastic containers. For each host plant latitude, longitude, and elevation data at the collection site were recorded using a GPS, and two samples with leaves, flowers, and fruits as available were collected for identification and vouchering. Fruits were dissected to obtain larvae. Of the total third instars, 25–50% were preserved in 70% ethanol for morphological study and DNA extraction, and the other subset of 50–75% of larvae were saved for rearing to the adult stage. Living larvae were killed by immersion in boiling water for 2 min, allowed to cool at room temperature for 2–5 min, then preserved in 5 ml vials with 70% ethanol. Rearing was conducted by placing the third instars into 1-liter plastic containers with a layer of 2.5–5.0 cm of moist vermiculite as a substrate for pupation. The tops of the containers were covered with a thin mesh of polyester or nylon fabric. Rearing containers were kept at room temperature, inspected daily, and the substrate was moistened if necessary. Reared adults were kept alive for 24–48 h to allow full development of coloration, then killed and preserved in 95% ethanol. Before females hardened in alcohol, the aculeus was extruded for identification.

### ﻿Identification of flies and host plants

Reared adults were identified by ALN and EJR. Vouchers are deposited at the Florida State Collection of Arthropods (**FSCA**), Gainesville, Florida, USA; U.S. National Museum of Natural History, Smithsonian Institution, Washington, DC (**USNM**); and Museo de Historia Natural Javier Padro, Universidad Nacional Mayor de San Marcos, Lima, Peru (**MHNJP**). Host plants were identified by Juan Celidonio Ruiz Herbarium Amazonense (**AMAZ**), Rufo Bustamante (Asociación para la Conservación de la Cuenca Amazónica - ACCA), Milton Zambrano (Pontificia Universidad Católica del Ecuador – PUCE, Estacion Cientifica Yasuni), and Sabitrie Jairam-Doerga (Nationaal Herbarium van Suriname – BBS). Plant vouchers are deposited at the U.S. National Museum of Natural History, Smithsonian Institution, Washington, D.C. (**USNM**); Universidad Nacional San Antonio Abad de Cusco, Perú (**UNSAAC**); Herbarium Amazonense (**AMAZ**) of the Universidad Nacional de la Amazonia Peruana, Iquitos, Perú (**UNAP**); and Nationaal Herbarium van Suriname (**BBS**).

### ﻿Specimen preparation

Intact preserved larvae were submerged in 70% ethanol to photograph the habitus (dorsal and lateral views) at 10 × magnification, and anal lobe and oral ridges at 150 × magnification using a Zeiss Discovery V12 dissecting microscope, Zeiss AxioCam ICc 5 digital camera and ZEN 2 software (Blue edition 2011). For slide-mounted specimens, the cephaloskeleton was detached from the head, and the cuticle was incised following Steck et al. (1990).

### ﻿Preparation and imaging of slide-mounted larval specimens

Internal tissues (gut and muscle tissue) were placed in 95–100% ethanol for molecular analysis. The cuticle of specimens to be slide-mounted was macerated overnight in 10% cold sodium hydroxide solution (NaOH) and cleaned by washing with distilled water and squeezing out the undigested internal tissues with an insect pin. Then the cephaloskeleton and cuticle were separately slide mounted in glycerin for observation and imaging using a Zeiss Axio Imager M2 compound microscope, Zeiss AxioCam 503 color digital camera and ZEN 2 software. The cephaloskeleton was photographed and measured at 100 ×, and the prothoracic and posterior spiracles at 400 ×. Measurements were taken as described in Steck and Wharton (1988). Stacks of images were rendered with the Z-stack function of ZEN 2 and Zerene Stacker software. After imaging, specimens were returned to 70% ethanol for permanent preservation and stored at FSCA.

### ﻿Preparation and imaging of larval specimens for SEM

The fifth abdominal segment was removed and placed in 95–100% ethanol for DNA extraction. The remaining anterior and posterior ends of the specimens were dehydrated by passing through an ethanol series of 70, 80, 95, and 100% (1 h each), followed by ethyl acetate (1 or 2 h), then air-dried, individually mounted on stubs with carbon tape, placed in a desiccator overnight, and sputter-coated with gold-palladium. Stub-mounted specimens were photographed and examined with a Phenon XL G1 and G2 Desktop SEM (Nanoscience Instruments, ThermoFischer Scientific, Phoenix, Arizona, USA) (Figs 28–32, 36, 39, 41–44, and 48–51) and JEOL JSM–5510LV SEM (JEOL USA, Inc., Peabody, Massachusetts, USA) (other SEM figures) at FDACS/DPI, Gainesville, FL. After imaging, specimens were stored at FSCA.

### ﻿DNA barcodes for confirmation of larval identity

DNA was extracted from adult and larval specimens using Qiagen DNeasy Blood and Tissue kits. COI barcodes were amplified using the primers LCO1490/HCO2198 (Folmer et al. 1994) or LEPF1/LEPR1 (Hebert et al. 2004). Alternatively, the primers C1-J-1632 (Kambhampati and Smith 1995) and C1-N-2191 (Simon et al. 1994) were used in some samples. PCR products were purified and bidirectionally sequenced on an Applied Biosystems SeqStudio Platform with BigDye Terminator v. 3.1 chemistry. Sequence traces were trimmed and assembled in Sequencher 5.4.6. K2P sequence similarity (Kimura 1980) between adults and larvae was evaluated in MEGA7 (Kumar et al. 2016) and by GenBank BLAST searches. Larvae were identified with the consensus identity function in BarcodingR (Zhang et al. 2017) using a library of previously published and newly sequenced *Anastrepha*COI barcodes (Moore et al. in press). The consensus identity function in BarcodingR provides identifications of protein-coding sequences by three methods: fuzzy-set based, BP-based, and Bayesian methods (Munch et al. 2008; Zhang et al. 2008, 2012; Hao et al. 2011; Zhang et al. 2017). Consensus identifications are supported by numerical “votes”, which is the total of the three analyses that provided the same identification. A support of two or three votes was the criterion of confidence used to make a molecular identification. Comparisons between larval and identified adult sequences yielding a single vote were considered ambiguous.

### ﻿Terminology

**Figures 1–5.**
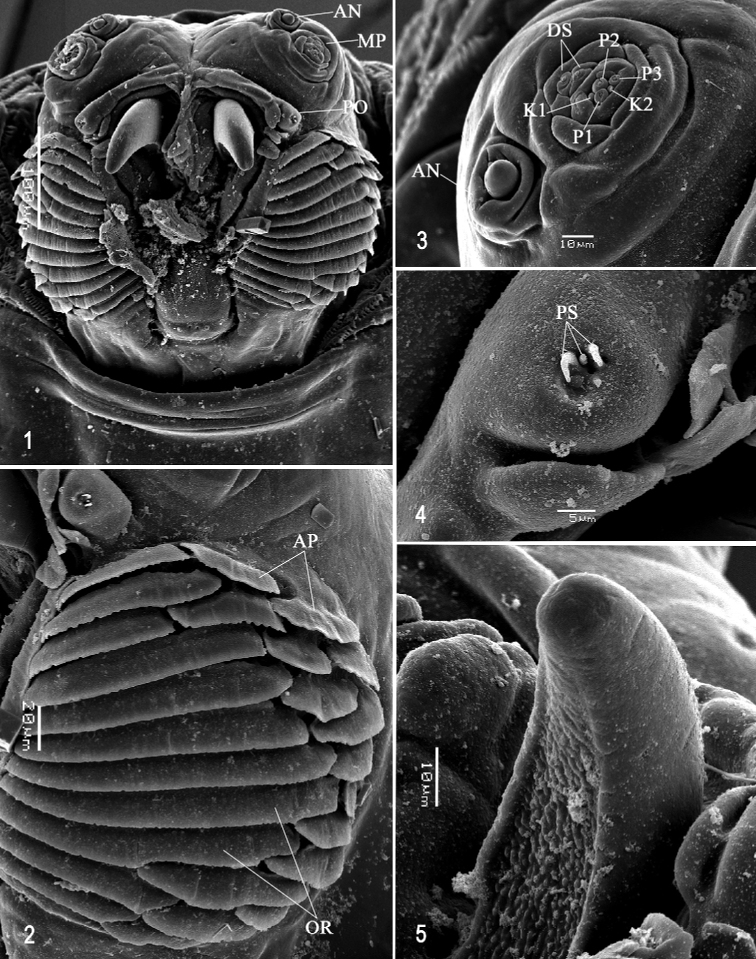
Scanning electron photomicrographs of third instar of *Anastrephaaphelocentema***1** pseudocephalon **2** oral ridges **3** antenna and maxillary palp **4** preoral organ **5** ventral surface of mouthhook. Abbreviations: AN, antenna; MP, maxillary palp; PO, preoral organ; AP, accessory plates; OR, oral ridges; P1–P3, papilla sensilla; K1, K2, knob sensilla; DS, dorsolateral papilla sensilla; PS, peg sensilla. Scale bars: 5 μm (**4**); 10 μm (**3, 5**); 20 μm (**2**); 100 μm (**1**).

We largely follow the terminology used in previous *Anastrepha* larva descriptions (e.g., Steck and Wharton 1988; White et al. 1999; Carroll et al. 2004; Rodriguez et al. 2021) and adopt current usage in other Diptera families and understanding of homologies following Courtney et al. (2000) and Borkent and Sinclair (2017). Terminology of the sensilla of the maxillary palp and dorsolateral group follows Chu-Wang and Axtell (1972). In the present study we introduce the term vertical comb-like process (Figs 53, 68, 111), which are elongate structures, connected laterally at the oral margin and projecting medially into the oral cavity, located adjacent to the labium and posterior to the oral ridges. We also introduce the term fringed and redefine the nomenclature of serrate, emarginate, and dentate to describe the posterior margins of the oral ridges and accessory plates. An emarginate margin is defined as having rounded (inverted U) or triangular (inverted V) notches, one quarter to one third the width of the basal part of the oral ridge, that are widely spaced (see White and Elson-Harris 1992: pls 5a, 6b; Carroll et al. 2004; Rodriguez et al. 2021: fig. 2). A serrate margin is defined as having small or minute teeth or projections less than one quarter the width of the basal part of the oral ridge, that are closely or moderately spaced (Figs 1, 2). A dentate margin is defined as having toothlike projections, one quarter to half the width of the basal part of the oral ridge, that are closely or moderately spaced, of even or uneven length, usually both sides of each tooth are equal, and the acute tips are above the middle of base (Figs 42, 109, and 110 for oral ridges; see Fig. 15 for accessory plates). A fringed margin is defined as having filaments or projections at least as long as the basal, non-incised part of the oral ridge (Figs 29, 67, 81, 82, 84, 95, 96), that are closely or moderately spaced, usually of even length, and tapering to a blunt tip.

### ﻿Larval specimens of the outgroup for comparative morphology of the pseudocephalon

Larvae of 13 *Anastrepha* species classified in eight species group were photographed and examined with a JEOL JSM–5510LV SEM (JEOL USA, Inc., Peabody, Massachusetts, USA) at FDACS/DPI, and the descriptions and illustrations of 15 species in the literature were consulted (Table 1). After imaging, specimens were stored at FSCA. Characters of the pseudocephalon with relevant phylogenetic signal were evaluated to construct a character matrix including the ingroup (*mucronota* species group) and outgroup taxa.

**Table 1.** List of the examined outgroup taxa including literature for coding larval characters of the pseudocephalon.

Species group	Species	Collection site	Unique identifier	Additional specimens from literature
*curvicauda*	*A.curvicauda*	USA: Florida: Miami Dade Co., Homestead area, reared from fruit of *Caricapapaya* L.	FF20170329.06– FF20170329.15	Frías et al. 2006
*fraterculus*	*A.amita*	–	–	Dutra et al. 2018a
	*A.amplidentata*	Peru: Madre de Dios: Puerto Maldonado, Centro de Investigación y Capacitación Rio Los Amigos, 12.5713°S, 70.0905°W, 277 m	AP20170713.09, AP20170713.12, AP20170713.15, AP20170713.17	Rodriguez et al. 2021
	*A.bahiensis*	Peru: Cusco: Pilcopata, Centro de Investigación Villa Carmen, 12.9020°S, 71.4113°W, 765 m.	AP20171024.08– AP20171024.10	Dutra et al. 2012
	*A.coronilli*	Panama: Cocle: Villa Carmen Village, 8.7973°S, 80.5470°W, 76 m.	AP20171115.01– AP20171115.03	Dutra et al. 2012
	*A.durantae*	Peru: Cusco: Echarate, Manto Real, 12.6552°S, 72.5766°W, 770 m.	AP20190827.16– AP20190827.18	Rodriguez et al. 2021
	*A.ludens*	Panama: Cocle: Barreta, 8.5892°S, 80.7139°W, 546 m. USA: Texas: USDA, ARS Lab. colony.	AP20180703.06– AP20180703.15	Carroll and Wharton 1989
	*A.sororcula*	–	–	Dutra et al. 2018a
	*A.suspensa*	–	–	White and Elson-Harris 1992
	*A.zenildae*	–	–	Dutra et al. 2018a
*grandis*	*A.grandis*	Austria: Viena: Seibersdorf, IAEA colony. USA: Florida: infested commodity from Peru intercepted at Miami International Airport	AP20180109.04– AP20180109.13	–
*leptozona*	*A.leptozona*	Peru: Cusco: Pilcopata, Centro de Investigación Villa Carmen, 12.8946°S, 71.4112°W, 619 m.	AL-01–AL-13	Frías et al. 2009
*pseudoparalella*	*A.limae*	Panama: Cocle: Villa Carmen Village, 8.7988°S, 80.5509°W, 77 m.	AP20180524.10– AP20180524.19	–
*serpentina*	*A.pulchra*	Peru: Madre de Dios: Puerto Maldonado, Centro de Investigación y Capacitación Rio Los Amigos, 12.5554°S, 70.1091°W, 281 m.	APU-01–APU-09	Dutra et al. 2018b
	*A.serpentina*	Peru: Lima: SENASA Lab. UCPMF/DM, colony	ASR-01–ASR-08	White and Elson-Harris 1992
*spatulata*	*A.pickeli*	Panama: Cocle: Barreta, 8.5812°S, 80.7414°W, 735 m. Peru: Cusco: Pilcopata, Centro de Investigación Villa Carmen, 12.5342°S, 71.2410°W, 534 m	API-01–API-12	Dutra et al. 2018b
*striata*	*A.striata*	Peru: Cusco: Pilcopata, Centro de Investigación Villa Carmen, 12.8936°S, 71.4054°W, 536 m.	AP20160223.01– AP20160223.02, AP20160223.06– AP20160223.07, AP20160223.09, AP20160223.11	White and Elson-Harris 1992

### ﻿Visualization of the phylogenetic relationships within the *mucronota* group

The novel larval morphological character states of the pseudocephalon were plotted on the phylogenetic tree for *Anastrepha* from Mengual et al. (2017: fig. 1) to aid our discussion of relationships within the *mucronota* group. We redrew a section of the phylogeny with the aid of FigTree v. 1.4.4 (Rambaut 2018), and the image (Fig. 123) was modified using Adobe Illustrator and Adobe Photoshop Elements.

## ﻿Results

### ﻿Descriptions of third instars

#### ﻿ Anastrepha aphelocentema

Taxon classificationAnimaliaDipteraTephritidae

Stone, 1942

AD80565F-5192-593D-AD34-78EDDAD1142A

Figs 1–5 , 6–11 , 12–13

##### Material examined.

Mexico • 4 larvae; Veracruz, Xalapa, Papantla; 20.3992°N, 97.3469°W; 72 m a.s.l.; Jul.1998; M. Aluja leg.; reared from fruit of *Pouteriaglomerata* (Miq.) Radlk. (Sapotaceae); FSCA (AP20171024.07, AP20190827.04, AP20180726.01–AP20180726.02).

##### Diagnosis.

*Anastrephaaphelocentema* runs to *A.leptozona* Hendel in the key of Steck et al. (1990), and to two species (*A.leptozona* and *A.serpentina*) in that of Carroll et al. (2004). It differs from all species within the *mucronota* group in having the posterior margins of the oral ridges and accessory plates finely serrate or entire. In addition, *A.aphelocentema* can be separated from *A.curvicauda* (Gerstaecker) by the position of the preoral organ (lateral vs. anterior to the mouthhook), and from *A.curitis* Stone in having a higher number of oral ridges (12–14 vs. 8–11). It can be also distinguished from most other species for which larvae have been described by the number of tubules of the prothoracic spiracle (24–27). This includes larvae of *A.pallens* Coquillett of the *daciformis* group (17–22 tubules), various species of the *fraterculus* group (9–22; see Rodriguez et al. 2021), *A.grandis* (Macquart) of the *grandis* group (31–37), *A.leptozona* of the *leptozona* group (15–21), two species of the *pseudoparallela* group (*A.limae* Stone with 18–21, and *A.consobrina* (Loew) with 12–15), two species of the *spatulata* group (*A.pickeli* Lima with 16–23, and *A.interrupta* Stone 10–13), two species of the *serpentina* group (*A.pulchra* Stone with 18–23, and *A.serpentina* (Wiedemann) with 13–19), and two species of the *striata* group (*A.bistrigata* Bezzi with 13–20, and *A.striata* Schiner with 11–18). The larva of *Anastrephasagittata* Stone (*dentata* group), reared from seeds of the related species *Pouteriacampechiana* (Kunth) Baehni, was described with limited data (Baker et al. 1944) but can be morphologically separated from *A.aphelocentema* by the longer and narrower posterior spiracle openings.

##### Description.

***Habitus*.** Third instar elongate, cylindrical, tapered anteriorly and truncate posteriorly; color creamy; amphipneustic. Length 11.00‒11.77 mm and width 2.03‒2.12 mm at the sixth abdominal segment.

***Pseudocephalon*** (Figs 1–4). Antenna and maxillary palp on moderately developed lobe. Antenna with cylindrical base and apical knob. Maxillary palp bearing three papilla sensilla, two knob sensilla; dorsolateral group of sensilla bearing two well-developed papilla sensilla, aligned perpendicular to palp and surrounded by collar. Facial mask globular in lateral view. Preoral organ bearing three unbranched peg sensilla, located apically on simple elongate preoral lobe or on separate small cylindrical lobe (asymmetrical in Fig. 1) lateral to the mouthhook; three or four petal-like secondary lobes adjacent to preoral organ. Oral ridges in 12–14 rows, posterior margin finely serrate or entire; 15–17 accessory plates, posterior margin usually serrate, most oral ridges bordered with single accessory plate laterally, except anterior 2–5 plates in two series, plates much narrower than ridges. Labium triangular, anterior surface knobby (not clearly visible in Fig. 1), ventrally with two visible sensilla and tubercles.

**Figures 6–11.**
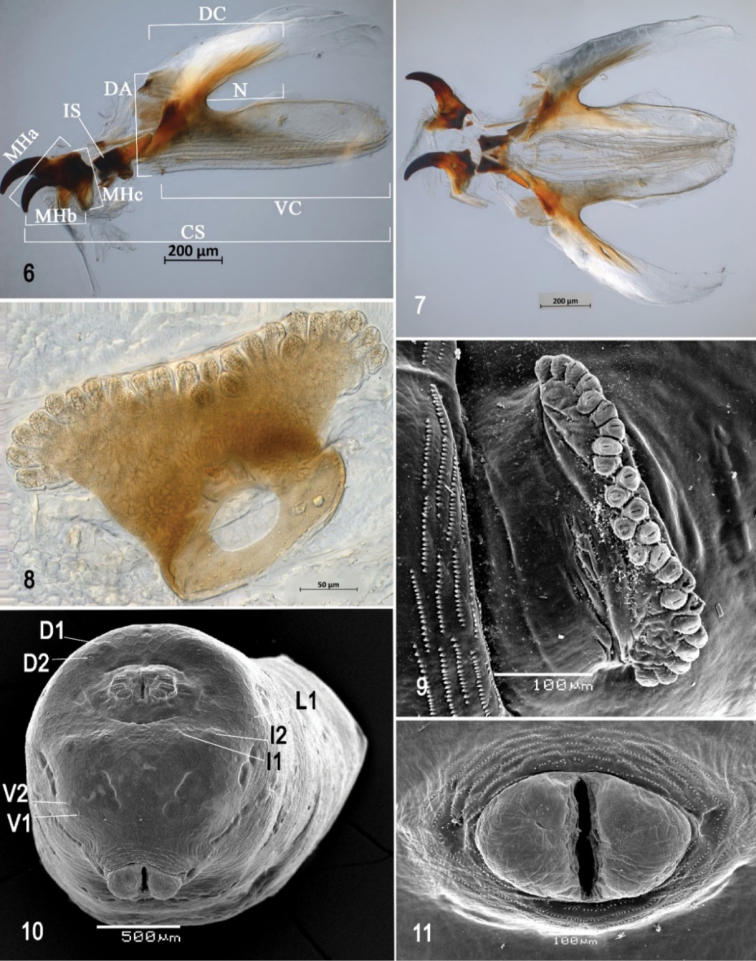
Optical photomicrographs and scanning electron photomicrographs of third instar of *Anastrephaaphelocentema***6** cephaloskeleton, lateral view **7** cephaloskeleton, dorsal view **8** prothoracic spiracle, lateral view **9** prothoracic spiracle, dorslateral view **10** caudal segment **11** anal lobe. Abbreviations: CS, total length of cephaloskeleton; MHa, mouthhook length a; MHb, mouthhook length b; MHc, mouthhook height c; IS, intermediate sclerite; DA, dorsal arch; DC, length of sclerotized area of dorsal cornu; N, notch; VC, length of ventral cornu; D1, D2, dorsal tubercles and sensilla; I1, I2, intermediate tubercles and sensilla; L1, lateral tubercle and sensillum; V1, V2, ventral tubercles and sensilla. Scale bars: 50 μm (**8**); 100 μm (**9, 11**); 200 μm (**6, 7**); 500 μm (**10**).

***Cephaloskeleton*** (Figs 5–7). Total length from tip of mouthhook to end of ventral cornu 1.31 mm. Mouthhook well sclerotized, black apically and basally; length a 0.28 mm; length b 0.22 mm; height c 0.20 mm; ratio a:b 1.29; ratio a:c 1.4. Tooth long, sharp, deeply concave ventrally, strongly curved, concave ventrally, ventral surface rough. Intermediate sclerite 0.22–0.23 mm long, 0.16 mm wide at ventral bridge. Epipharyngeal sclerite visible only in dorsal view, with medial lobe directed anteriorly. Labial sclerite robust, sclerotized in dorsal view. Parastomal bar extending for almost entire length of intermediate sclerite. Dorsal arch 0.35 mm high. Dorsal cornu with well-defined sclerotized area adjacent to notch, 0.50 mm long. Dorsal bridge prominently projecting anteriorly from dorsal cornu and slightly sclerotized. Anterior sclerite irregularly shaped and sclerotized. Cornu notch (N) 0.33 mm long and cornu notch index (N/DC) 0.7. Ventral cornu with poorly defined sclerotized area along edge of notch. Pharyngeal filter with weakly sclerotized anterior bar and eight ridges forming a series of grooves along length of ventral cornu. Ventral cornu 0.81 mm long from pharyngeal bar to posterior end of grooves. Ventral cornu 1.63 × as long as sclerotized area of dorsal cornu.

***Thoracic and abdominal segments*.** Thoracic segments with dorsal spinules conical, symmetrical to slightly posteriorly curved; dorsal spinule pattern, as follows: T1 with 5‒7 rows, forming scalloped plates; T2 with four or five rows; T3 lacking spinules; ventral spinule pattern as follows: T1 with 5‒7 rows; T2 with 0‒2 rows; T3 with two rows. Abdominal segments (A1–A8) lacking dorsal spinules; ventral creeping welts present on all abdominal segments; ventral spinule pattern as follows: A1 with six or seven rows; A2 with 10–12 rows; A3–A6 with 14–18 rows; A8 with 12–16 rows. Additional four or five discontinuous rows of spinules surrounding anal lobes, spinules all equally small, basally broad, distally sharply pointed, pointing away from anal lobes.

***Prothoracic spiracle*** (Figs 8, 9). Bilobed, bearing 24–27 tubules, distally rounded and arranged in a single, sinuous row laterally and double row medially. Spiracle distal width 0.35–0.36 mm; basal width 0.19 mm at junction with trachea.

***Caudal segment*** (Figs 10, 11). Dorsal tubercles and sensilla weakly developed, D1 distinctly anterior to D2. Intermediate tubercles (I1 and I2) moderately developed, I1 lateral and sometimes slightly dorsal to I2, associated sensilla weakly developed. Lateral (L1) tubercles, and associated sensilla weakly developed. Ventral (V1 and V2) tubercles and sensilla weakly developed, V1 distinctly posterior to V2. Anal lobe entire or grooved and moderately protuberant.

**Figures 12, 13.**
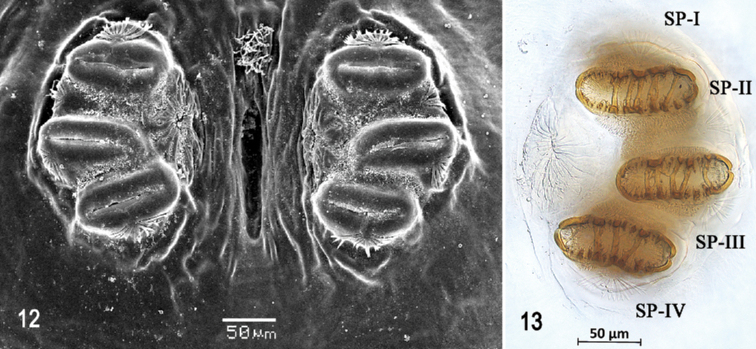
Scanning electron photomicrograph and optical photomicrograph of posterior spiracles of third instar of *Anastrephaaphelocentema*. Abbreviations: SP-I to SP-IV, spiracular processes. Scale bars: 50 μm (**12, 13**).

***Posterior spiracle*** (Figs 10, 12, 13). Located above horizontal midline. Posterior spiracle openings with thick rimae and numerous trabeculae; 94–101 µm long; 35‒37 µm wide; ratio length/width 2.68‒2.72. Ecdysial scar apparent. Felt chamber oval, 190‒191 µm in diameter at junction with trachea. Spiracular process SP-I comprising 4‒9 trunks and 12‒21 tips; ratio tips/trunks 2.3‒3.0; basal width 9‒12 µm; ratio basal width/length of spiracular opening 0.09‒0.12. SP-II comprising three or four trunks and seven or eight tips. SP-III comprising 3‒7 trunks and 6‒12 tips. SP-IV comprising 3‒7 trunks and 10‒15 tips; ratio tips/trunks 2.14‒3.33; basal width 9‒10 µm; ratio basal width/length of spiracular opening 0.09‒0.11.

##### Distribution.

*Anastrephaaphelocentema* is known only from Mexico (northern Veracruz and San Luis Potosí) (Aluja et al. 2000; Norrbom 2004; Hernández-Ortiz 2007; CoFFHI 2020).

##### Biology.

This species was reared from fruit of *Pouteriaglomerata*. It has been previously reared from fruits of *Casimiroaedulis* La Llave and Lex. (Rutaceae) (Hernández-Ortiz 1992) and *Pouteriaglomerata* (Sapotaceae) (Stone 1942; Baker et al. 1944; Norrbom and Kim 1988; Aluja et al. 2000; Hernández-Ortiz 2007).

##### Molecular identification.

COI barcodes were generated from four larvae and submitted to GenBank (MT644043, MT654963–MT654965). These data further confirm the identity of the described larvae. K2P distances between *A.aphelocentema* larvae and the available adult sequence (KY428328) were less than one percent. BLAST searches were consistent with our new data, yielding only one good match: *A.aphelocentema* (99.84% sequence identity; KY428328). Additionally, all four barcodes returned consensus identifications of *A.aphelocentema* with three votes using the identity function in BarcodingR (Moore et al. in press).

#### ﻿ Anastrepha caballeroi

Taxon classificationAnimaliaDipteraTephritidae

Norrbom, 2015

9E1C2ABD-61CC-5971-8C7B-0B7C5212416B

Figs 14–19 , 20–25 , 26–27

##### Material examined.

Peru • 13 larvae; Madre de Dios, Puerto Maldonado, Centro de Investigación y Capacitación Río Los Amigos (CICRA), trail 2; 12.5612°S, 70.1085°W; 287 m a.s.l.; 28 Jan. 2014; E. J. Rodriguez and J. Caballero leg.; reared from fruit of *Quararibeamalacocalyx* (A. Robyns and S. Nilsson) W.S. Alverson (Malvaceae); FSCA (AP20180321.05–AP20180321.14, AP20190827.07–AP20190827.09).

##### Diagnosis.

*Anastrephacaballeroi* can be distinguished from all other species of *Anastrepha* by the dentate posterior margins of its accessory plates; in other species of the *mucronota* group the margins of the oral ridges are serrate or mostly or entirely fringed (see Tables 2–4). It also differs from all other *Anastrepha* species in having 27‒36 accessory plates mostly in two series and covering a much larger area than the oral ridges.

**Table 2.** Diagnostic characters of the pseudocephalon of species within the *mucronota* group.

Species	Location of preroal organ	Shape of preoral lobe	Oral ridges		Accessory plates		Mouthhook		No. of comb-like processes
			Number	Posterior margins	Number	Posterior margins	Ventral surface	Length b (mm)	
*A.aphelocentema*	Lateral to MH	Long, narrow, with 3–4 petal-like lobes adjacent to preoral organ	12‒14	Finely serrate or entire	15‒17; mostly in one series	Finely serrate or entire	Concave, Rough	0.22	Absent
*A.caballeroi*	Anterolateral to MH	Long, narrow, split apically, extending posterior to preoral organ	14‒15	Entire or undulant	27‒36, covering a much larger area than oral ridges	Dentate	Concave, eroded	0.21–0.23	Absent
*A.crebra*	Anterolateral to MH	Long, broad, extending posterior to preoral organ	13‒15	Fringed	Present; apparently in one series	Fringed	Concave, medial carina, smooth	0.16–0.17	Absent
*A.haplacantha*	Lateral to MH	Long, narrow, with 3–5 single or bifid secondary lobes adjacent to preoral organ	19‒20	Dentate with long moderately spaced projections	Numerous plates	Fringed	Concave, apparently smooth	0.21–0.22	Absent
*A.korytkowskii*	Anterior to MH	Short, irregular-rounded lobe, smaller than preoral organ	12‒14	Irregularly dentate and entire	14‒20; plates in one series	Fringed	Concave, eroded	0.10‒0.13	3–5
*A.mucronota*	?	?	13‒15	?	?	?	?	?	?
*A.nolazcoae*	Anterior to MH	Short, narrow, extends to posterior middle of preoral organ	16‒19	Fringed, 3–4 posterior ridges entire	~ 36; medial and posterior plates in two series	Fringed	Concave, Smooth	0.12–0.15	6–8
*Anastrepha* sp. Peru-82	Anterior to MH	Short, broad, irregular shape, larger than preoral organ	22‒23	Densely fringed, posterior ridges dentate	Numerous plates, overlapping with oral ridges	Fringed	Concave, medial carina, smooth	0.18‒0.20	Absent
Anastrepha sp. nr.protuberans	Lateral to MH	Long, narrow	18‒23	Fringed	Present; apparently in one series	Fringed	Concave, smooth	0.24‒0.25	Absent
*Anastrepha* sp. Sur-16	Anterior to MH	Short-elongate, narrow, extends partially posterior to preoral organ	13‒16	Dentate with long closely spaced projections, 2–3 posterior ridges entire	Numerous plates; plates in two or more series	Fringed	Concave, eroded	0.16‒0.17	7–9

(?) Unknown data from previous studies.

**Table 3.** Diagnostic characters of the thoracic and abdominal segments of species within the *mucronota* group.

Species	Prothoracic spiracle		Dorsal spinule pattern	
	No. of tubules	Apical width (mm)	Thoracic segment	Abdominal segment
			No. of rows	No. of rows
*A.aphelocentema*	24‒27	0.35–0.36	T1 5‒7; T2 4‒5; T3 absent	Absent on A1‒A8
*A.caballeroi*	17‒21	0.28–0.33	T1 3; T2 3; T3 absent	Absent on A1‒A8
*A.crebra*	16‒21	0.22–0.24	T1 9‒11; T2 3‒5; T3 1‒2	Absent on A1‒A8
*A.haplacantha*	20‒24	0.32–0.35	T1 5‒7; T2 3‒4; T3 1	Absent on A1‒A8
*A.korytkowskii*	12‒18	0.19–0.24	T1 6‒7; T2 2‒5; T3 absent	Absent on A1‒A8
*A.mucronota*	20‒22	?	Present on T2‒T3 with minute spinules	?
*A.nolazcoae*	18‒21	0.26–0.34	T1 3‒5; T2 3‒5; T3 1‒2	Absent on A1‒A8
*Anastrepha* sp. Peru-82	23‒29	0.28–0.35	T1 2; T2 5‒6; T3 2‒3	A1 3; absent on A2–A8
Anastrephasp.nr.protuberans	22–30	0.41–0.44	T1 3; T2 4‒5; T3 4	A1 2; absent on A2–A8
*Anastrepha* sp. Sur-16	12‒17	0.23–0.28	T1 5; T2 3; T3 absent	Absent on A1‒A8

(?) Unknown data from previous studies.

**Table 4.** Diagnostic characters of the caudal segment of species within the *mucronota* group.

Species	Posterior spiracle (SP-I and SP-IV)				Anal lobe
	Length of spiracular opening (μm)	No. of trunks	No. of tips	Basal width (μm)	
*A.aphelocentema*	94–101	SP-I 4‒9; SP-IV 3‒7	SP-I 12‒21; SP-IV 10‒15	SP-I 9‒12; SP-IV 9‒10	Grooved, entire
*A.caballeroi*	76–89	SP-I 5‒8; SP IV 4‒7	SP I 10‒18; SP IV 7‒17	SP I 7‒13; SP IV 5‒7	Entire
*A.crebra*	58–73	SP-I 14‒18; SP IV 14‒20	SP I 33‒51; SP IV 31‒39	SP I 20‒30; SP IV 16‒28	Entire
*A.haplacantha*	69–80	SP-I 9‒12; SP IV 9‒12	SP I 13‒27; SP IV 16‒23	SP I 12‒18; SP IV 14‒15	Entire
*A.korytkowskii*	56–77	SP-I 9‒15; SP-IV 8‒15	SP-I 21‒33; SP-IV 17‒31	SP-I 14‒28; SP-IV 12‒21	Entire
*A.mucronota*	~100	SPI ~8‒9; SP IV ~7‒8	SPI 12; SP IV 11	?	?
*A.nolazcoae*	83–108	SP-I 8‒11; SP IV 4‒12	SP I 9‒26; SP IV 8‒24	SP I 9‒15; SP IV 7‒12	Entire
*Anastrepha* sp. Peru-82	84–97	SP- I 9‒11; SP-IV 7‒11	SP-I 12‒20; SP-IV 13‒16	SPI 12‒15; SP IV 9‒19	Entire
Anastrephasp.nr.protuberans	122–145	SP- I 5‒11; SP-IV 7‒10	SP-I 9‒20; SP-IV 14‒21	SPI 8‒11; SP IV 9‒12	Entire
*Anastrepha* sp. Sur-16	69–80	SP- I 13‒18; SP-IV 13‒17	SP-I 19‒34; SP-IV 25‒40	SPI 29‒36; SP IV 23‒34	Entire

(?) Unknown data from previous studies.

##### Description.

***Habitus*.** Third instar elongate, cylindrical, tapered anteriorly and caudal end truncate; color creamy; amphipneustic. Length 10.24‒10.61 mm and width 1.66‒1.69 mm at the sixth abdominal segment.

**Figures 14–19.**
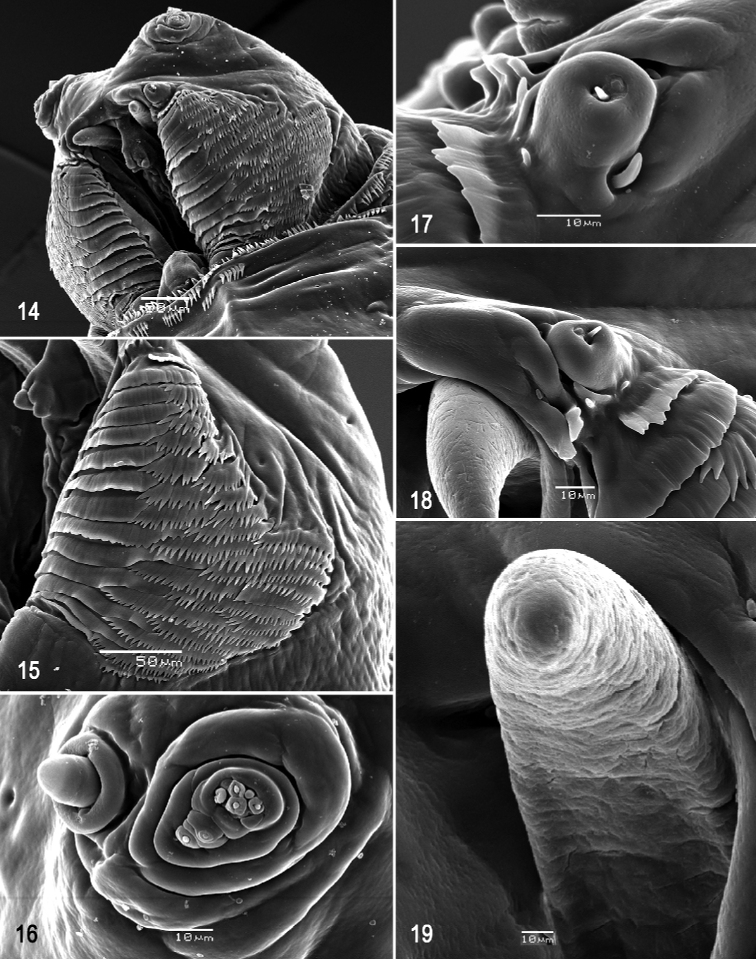
Scanning electron photomicrographs of third instar of *Anastrephacaballeroi***14** pseudocephalon **15** oral ridges **16** antenna and maxillary palp **17** preoral organ, dorsal view **18** preoral organ, dorsolateral view **19** ventral surface of mouthhook. Scale bars: 10 μm (**16–19**); 50 μm (**14, 15**).

***Pseudocephalon*** (Figs 14–18). Antenna and maxillary palp on moderately developed lobe. Antenna with cylindrical base and apical knob. Maxillary palp bearing three papilla sensilla, two knob sensilla; dorsolateral group of sensilla bearing two well-developed papilla sensilla, aligned perpendicular to palp and surrounded by collar. Facial mask partly globular in lateral view, upper right section lacking ridges and accessory plates and forming almost a right angle. Preoral organ bearing 1–3 unbranched peg sensilla, located apically on small cylindrical lobe anterolateral to mouthhook, with or without one or two adjacent finger-like lobes; preoral lobe elongate, split apically, extending posterior to preoral organ. Oral ridges in 14 or 15 short rows, posterior margin entire or undulant (occasionally 1–3 posterior ridges emarginate); 27–36 accessory plates, posterior margin deeply dentate with sharply pointed teeth, anterior and posterior plates in one series, medial plates in two series, plates covering much larger area than oral ridges. Labium triangular, anterior surface knobby (not clearly visible in Fig. 14), ventrally with two visible sensilla on small tubercles.

**Figures 20–25.**
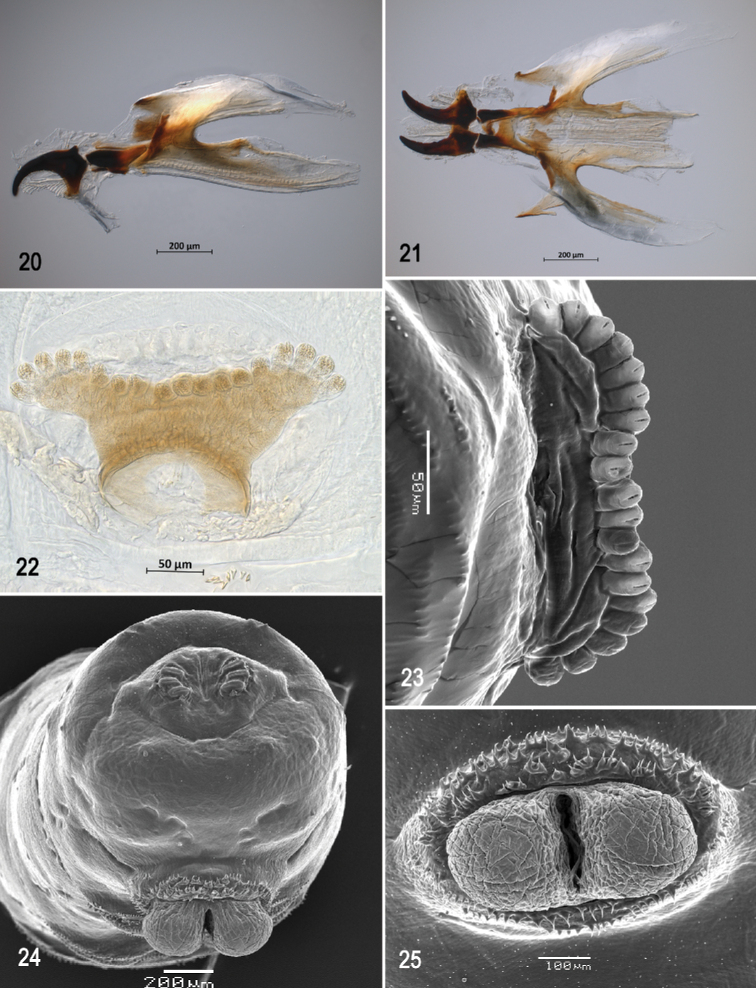
Optical photomicrographs and scanning electron photomicrographs of third instar of *Anastrephacaballeroi***20** cephaloskeleton, lateral view **21** cephaloskeleton, dorsal view **22** prothoracic spiracle, lateral view **23** prothoracic spiracle, dorsolateral view **24** caudal segment **25** anal lobe. Scale bars: 50 μm (**22, 23**); 100 μm (**25**); 200 μm (**20, 21, 24**).

***Cephaloskeleton*** (Figs 19–21). Total length from tip of mouthhook to end of ventral cornu 1.26–1.31 mm. Mouthhook well sclerotized, black apically and basally; length a 0.28–0.29 mm; length b 0.21–0.23 mm; height c 0.18–0.20 mm; ratio a:b 1.28–1.37; ratio a:c 1.45–1.63. Tooth long, sharp, strongly curved, concave ventrally, ventral surface eroded. Intermediate sclerite 0.21–0.23 mm long, 0.13–0.15 mm wide at ventral bridge. Epipharyngeal sclerite visible only in dorsal view, with medial lobe directed anteriorly. Labial sclerite short, robust, sclerotized in dorsal view. Parastomal bar extending for almost entire length of intermediate sclerite. Dorsal arch 0.27–0.29 mm high. Dorsal cornu with well-defined sclerotized area adjacent to notch, 0.51–0.54 mm long. Dorsal bridge prominently projecting anteriorly from dorsal cornu and slightly sclerotized. Anterior sclerite irregularly shaped and sclerotized. Cornu notch (N) 0.30–0.34 mm long and cornu notch index (N/DC) 0.59–0.63. Ventral cornu with poorly defined sclerotized area. Pharyngeal filter with weakly sclerotized anterior bar and seven ridges forming a series of grooves along length of ventral cornu. Ventral cornu 0.79–0.83 mm long from pharyngeal bar to posterior end of grooves. Ventral cornu 1.54–1.56 × as long as sclerotized area of dorsal cornu.

***Thoracic and abdominal segments*.** Thoracic segments with dorsal spinules conical, symmetrical to slightly curved posteriorly; dorsal spinule pattern in rows as follows: T1 with three rows, forming scalloped plates; T2 with three rows; T3 lacking spinules; ventral spinule pattern as follows: T1 with seven or eight rows; T2 with three rows; T3 with 0–2 rows. Abdominal segments (A1–A8) lacking dorsal spinules; ventral creeping welts present on all abdominal segments; ventral spinule pattern as follows: A1 with two or three rows; A2 with six or seven rows; A3 with seven or eight rows; A4–A5 with 7–9 rows; A6 with seven or eight rows, A7–A8 with six or seven rows. Additional three or four anterior and posterior discontinuous rows of spinules, and one or two lateral rows around anal lobes, spinules large, conical, distally sharp, pointing away from anal lobes.

***Prothoracic spiracle*** (Figs 22, 23). Bilobed, bearing 17–21 tubules, distally rounded and arranged in a single sinuous row. Spiracle distal width 0.28–0.33 mm; basal width 0.13‒0.16 mm at junction with trachea.

***Caudal segment*** (Figs 24, 25). Dorsal tubercles and sensilla well developed, D1 distinctly anterior to D2. Intermediate tubercles (I1 and I2) moderately developed, I1 lateral and sometimes slightly ventral to I2, associated sensilla weakly developed. Lateral (L1) and ventral (V1 and V2) tubercles, and associated sensilla weakly developed. Anal lobe entire and moderately protuberant.

**Figures 26, 27.**
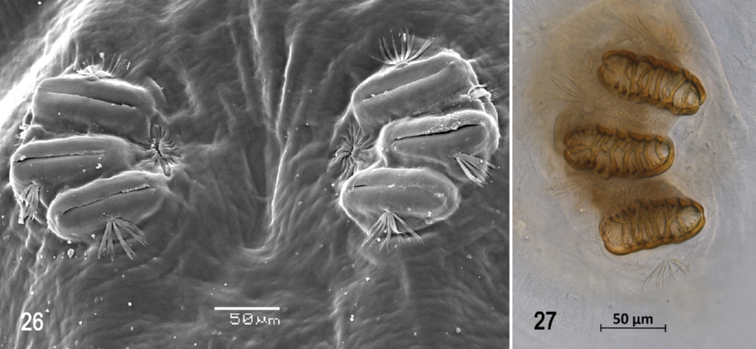
Scanning electron photomicrograph and optical photomicrograph of posterior spiracles of third instar of *Anastrephacaballeroi*. Scale bars: 50 μm (**26, 27**).

***Posterior spiracle*** (Figs 24, 26, 27). Located above horizontal midline. Posterior spiracle openings with thick rimae and numerous trabeculae; 76–89 µm long; 31‒37 µm wide; ratio length/width 2.4‒2.5. Ecdysial scar apparent. Felt chamber oval, 143‒184 µm in diameter at junction with trachea. Spiracular process SP-I comprising 5‒8 trunks and 10‒18 tips; ratio tips/trunks 2.0‒2.3; basal width 7‒13 µm; ratio basal width/length of spiracular opening 0.08‒0.15. SP-II comprising 3‒5 trunks and 3‒10 tips. SP-III comprising 4‒7 trunks and 4‒12 tips. SP-IV comprising 4‒7 trunks and 7‒17 tips; ratio tips/trunks 1.8‒2.4; basal width 5‒7 µm; ratio basal width/length of spiracular opening 0.06‒0.08.

##### Distribution.

*Anastrephacaballeroi* is known only from southeastern Peru (Cusco and Madre de Dios).

##### Biology.

We reared this species from fruit of *Quararibeamalacocalyx*, the only known host plant (Norrbom et al. 2015). The larvae feed only on the pulp of the fruit.

##### Molecular identification.

COI barcodes were generated from 13 larvae and nine adults of *A.caballeroi* and submitted to GenBank (MH070125, MT644046–MT644048, MT654994–MT655010, MT763935). One additional adult sequence was available for analysis (KY428405). These data further confirm the identity of the described larvae. K2P distances between *A.caballeroi* individuals ranged from 0.0–1.6%. In our larger COI dataset for *Anastrepha*, *A.caballeroi* is nearest-neighbor to the undescribed *Anastrepha* sp. Yasuni 01 from Ecuador. One of the *A.caballeroi* barcodes (MH070125) is more similar to A. sp. Yasuni 01 than other *A.caballeroi*. However, all barcoded larval specimens of *A.caballeroi* are best matches to adult *A.caballeroi* sequences. BLAST searches were consistent with our new data, yielding only two good matches, both to *A.caballeroi* (98.07%–100% sequence identity; KY428405 and MH070125). Additionally, all thirteen larval barcodes returned consensus identifications of *A.caballeroi* with three votes (Moore et al. in press).

#### ﻿ Anastrepha crebra

Taxon classificationAnimaliaDipteraTephritidae

Stone, 1942

D651CF71-9C2D-5B75-BBD2-31E2E795D4F2

Figs 28–32 , 33–38 , 39–40

##### Material examined.

Peru • 4 larvae; Madre de Dios, Puerto Maldonado, Centro de Investigación y Capacitación Río Los Amigos (CICRA), trail 21; 12.5721°S, 70.0889°W; 232 m a.s.l.; 22 Mar. 2016; N. Zenteno leg.; reared from fruit of *Quararibeawittii* K. Schumann and O. Ulbrich (Malvaceae); FSCA (AP20180315.6–AP20180315.10, AP20180329.08, AP20210415.01).

##### Diagnosis.

*Anastrephacrebra* can be distinguished from other species of *Anastrepha*, except *A.nolazcoae*, *Anastrepha* sp. Peru-82, and Anastrephasp.nr.protuberans, by the fringed posterior margin of its oral ridges. *Anastrephacrebra* differs from the latter three species in having fewer oral ridges, a higher number of trunks and tips of the posterior spiracular processes, and shorter spiracular opening length on the posterior spiracle (see Tables 2–4).

##### Description.

***Habitus*.** Third instar elongate, cylindrical, tapered anteriorly and caudal end truncate; color creamy; amphipneustic. Length 6.83‒7.36 mm and width 1.10‒1.21 mm at the sixth abdominal segment.

**Figures 28–32.**
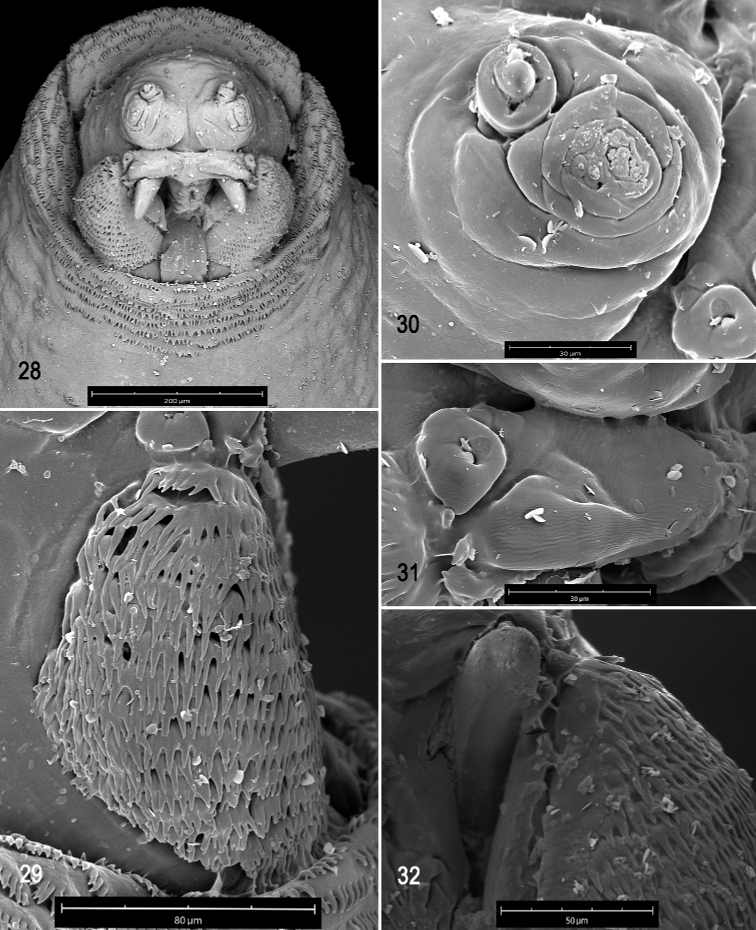
Scanning electron photomicrographs of third instar of *Anastrephacrebra***28** pseudocephalon **29** oral ridges **30** antenna and maxillary palp **31** preoral organ **32** ventral surface of mouthhook. Scale bars: 30 μm (**30, 31**); 50 μm (**32**); 80 μm (**29**); 200 μm (**28**).

***Pseudocephalon*** (Figs 28–31). Antenna and maxillary palp on moderately developed lobe. Antenna with cylindrical base and apical knob. Maxillary palp bearing three papilla sensilla, two knob sensilla; dorsolateral group of sensilla bearing two well-developed papilla sensilla, aligned at oblique angle to palp and surrounded by collar. Facial mask globular in lateral view. Preoral organ bearing one peg sensillum, located apically on small cylindrical lobe anterolateral to the mouthhook, with two or three adjacent irregular secondary lobes; preoral lobe elongate, broad, extending slightly posterior to preoral organ. Oral ridges in 13–15 rows, posterior margins fringed; accessory plates apparently in one series lateral to oral ridges covering a much smaller area than oral ridges, with fringed posterior margins. Labium narrow, surface channeled medially, ventrally with two visible sensilla on small tubercles.

**Figures 33–38.**
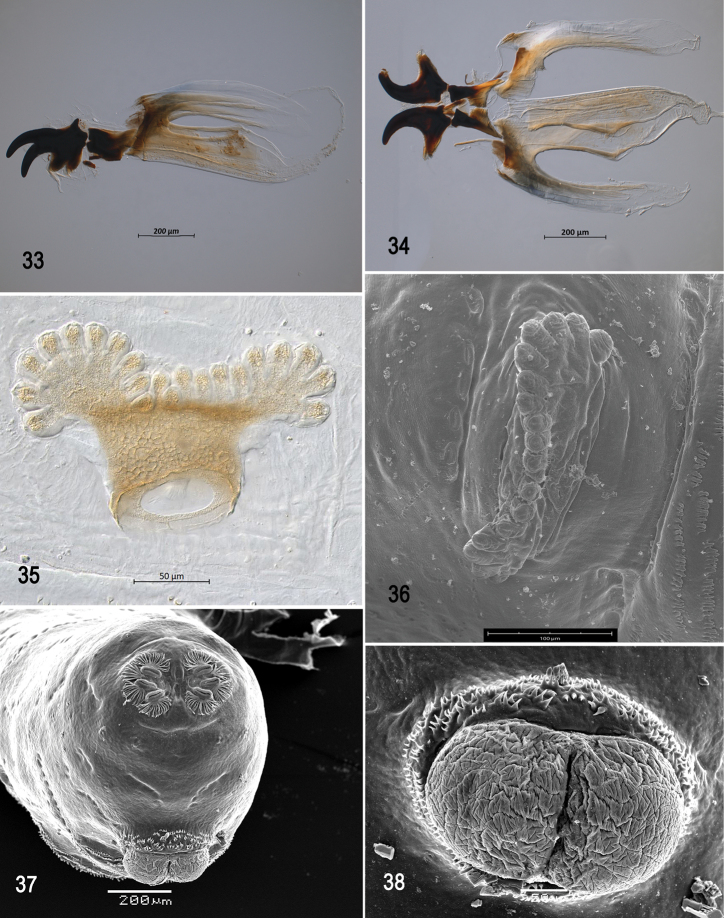
Optical photomicrographs and scanning electron photomicrographs of third instar of *Anastrephacrebra***33** cephaloskeleton, lateral view **34** cephaloskeleton, dorsal view **35** prothoracic spiracle, lateral view **36** prothoracic spiracle, dorsolateral view **37** caudal segment **38** anal lobe. Scale bars: 50 μm (**35, 38**); 100 μm (**36**); 200 μm (**33, 34, 37**).

***Cephaloskeleton*** (Figs 32–34). Total length from tip of mouthhook to end of ventral cornu 1.08–1.13 mm. Mouthhook well sclerotized, black apically and basally; length a 0.23–0.29 mm; length b 0.16–0.17 mm; height c 0.16–0.20 mm; ratio a:b 1.44–1.71; ratio a:c 1.44–1.45. Tooth long, sharp, strongly curved, concave ventrally with medial carina, ventral surface smooth. Intermediate sclerite 0.18–0.20 mm long, 0.14 mm wide at ventral bridge. Epipharyngeal sclerite visible only in dorsal view, with medial lobe directed anteriorly. Labial sclerite robust, sclerotized, and triangular in dorsal view. Parastomal bar extending three-fourths length of intermediate sclerite. Dorsal arch 0.23–0.24 mm high. Dorsal cornu with well-defined sclerotized area adjacent to notch, 0.42–0.48 mm long. Dorsal bridge projecting anteriorly from dorsal cornu and sclerotized. Anterior sclerite irregularly shaped and sclerotized. Cornu notch (N) 0.36 mm long and cornu notch index (N/DC) 0.75–0.85. Ventral cornu with weakly defined sclerotized area. Pharyngeal filter with weakly sclerotized anterior bar and ridges forming a series of grooves along length of ventral cornu. Ventral cornu 0.62–0.65 mm long from pharyngeal bar to posterior end of grooves. Ventral cornu 1.4–1.5 × as long as sclerotized area of dorsal cornu.

***Thoracic and abdominal segments*.** Thoracic segments with dorsal spinules conical, symmetrical to slightly curved posteriorly; dorsal spinules pattern in rows as follows: T1 with 9‒11 rows, forming scalloped plates; T2 with 3‒5 rows; T3 with one or two rows; ventral spinule pattern as follows: T1 with 11–15 rows; T2 and T3 lacking spinules. Abdominal segments (A1–A8) lacking dorsal spinules; ventral creeping welts present on all abdominal segments; ventral spinule pattern as follows: A1 with four rows; A2 with 8–10 rows; A3 with 10–13 rows; A4 with 12 rows; A5 with 11–13 rows; A6 with 11 or 12 rows, A7 with 9–11 rows; A8 with nine or ten rows. Additional three anterior and posterior and two lateral irregular rows of spinules surrounding anal lobes, spinules large, conical, distally sharp, pointing away from anal lobes.

***Prothoracic spiracle*** (Figs 35, 36). Bilobed, bearing 16–21 tubules, distally rounded and arranged in a single sinuous row. Spiracle distal width 0.22–0.24 mm; basal width 0.09‒0.10 mm at junction with trachea.

***Caudal segment*** (Figs 37, 38). Dorsal (D1 and D2) tubercles and sensilla moderately developed; D1 distinctly anterior to D2. Intermediate tubercles I1 and I2 and associated sensilla moderately developed; I1 ventral to I2. L1, V1, and V2 tubercles and associated sensilla weakly developed. Anal lobe entire and protuberant.

**Figures 39–40.**
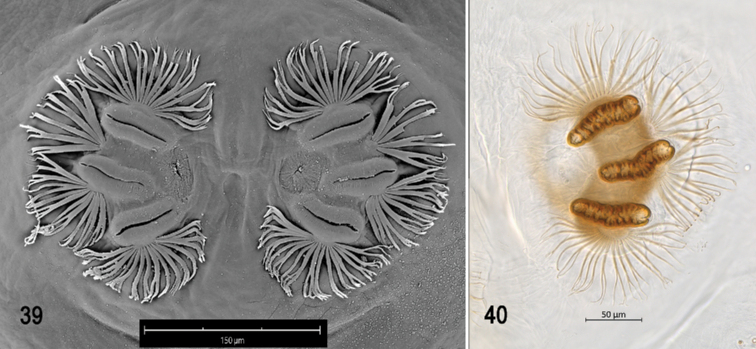
Scanning electron photomicrograph and optical photomicrograph of posterior spiracles of third instar of *Anastrephacrebra*. Scale bars: 50 μm (**40**); 150 μm (**39**).

***Posterior spiracle*** (Figs 37, 39, 40). Located above horizontal midline. Posterior spiracle openings with thick rimae and numerous trabeculae; 58–73 µm long; 21‒25 µm wide; ratio length/width 2.8‒2.9. Ecdysial scar apparent. Felt chamber oval, 127‒135 µm in diameter at junction with trachea. Spiracular process SP-I comprising 14‒18 trunks and 33‒51 tips; ratio tips/trunks 2.4‒ 2.8; basal width 20‒30 µm; ratio basal width/length of spiracular opening 0.33‒0.41. SP-II comprising 5‒7 trunks and 11‒23 tips. SP-III comprising 8‒13 trunks and 21‒32 tips. SP-IV comprising 14‒20 trunks and 31‒39 tips; ratio tips/trunks 2.0‒2.2; basal width 16‒28 µm; ratio basal width/length of spiracular opening 0.28‒0.39.

##### Distribution.

*Anastrephacrebra* is known from Mexico, Guatemala, Nicaragua, Costa Rica, Panama, Ecuador (Norrbom 2004; CoFFHI 2020), and Colombia (Rodríguez Clavijo et al. 2018). It is recorded for the first time from Peru.

##### Biology.

This species was reared from fruit of *Quararibeawittii*, a new host plant record for *A.crebra*. It has been previously reared from fruits of *Quararibeaasterolepis* Pittier (Malvaceae) (Stone 1942), *Quararibeafunebris* (La Llave) Vischer (Malvaceae) (Hernández-Ortiz and Pérez-Alonso 1993; Aluja et al. 2000), and *Quararibeayunckeri* Standl. (Malvaceae) (Aluja et al. 2003).

##### Molecular identification.

COI barcodes were generated from three larvae and three adults submitted to GenBank (MT655069–MT655074). These data further confirm the identity of the described larvae. K2P distances among *A.crebra* larvae and the 14 available adult sequences (KY428335, MK758576, MK758598, MK759164, MK759601, MK767247, MK767700, MK768011, MK768248, MK768483, MK769383, MK770033, MT655069–MT655071) ranged from 0.0–3.0%. BLAST searches were consistent with our new data, yielding good matches only to *A.crebra* (97.00–100.00% sequence identity). Additionally, all three larval barcodes returned consensus identifications of *A.crebra* with three votes (Moore et al. in press).

#### ﻿ Anastrepha haplacantha

Taxon classificationAnimaliaDipteraTephritidae

Norrbom & Korytkowski, 2012

CF13AB06-BFBA-5FDA-A6FE-45C341C271FC

Figs 41–44 , 45–50 , 51–52

##### Material examined.

Ecuador • 4 larvae; Orellana, Estacion Cientifica Yasuní, trail 5; 0.6692°S, 76.4018°W; 235 m a.s.l.; 9 Mar. 2018; E. J. Rodriguez leg.; reared from fruit of *Quararibeamalacocalyx*; FSCA (AP20200622.01–AP20200622.04).

##### Diagnosis.

*Anastrephahaplacantha* can be distinguished from other species of *Anastrepha*, except *A.korytkowskii* and *Anastrepha* sp. Sur-16, by the dentate posterior margin of its oral ridges. *Anastrephahaplacantha* differs from the latter two species in having more oral ridges, lacking comb-like processes, and by other morphological characters, such as number of trunks and tips of the posterior spiracular processes and basal width of the posterior spiracle (see Tables 2–4).

##### Description.

***Habitus*.** Third instar elongate, cylindrical, tapered anteriorly and caudal end truncate; color creamy; amphipneustic. Length 7.58‒8.31 mm and width 1.04‒1.42 mm at the sixth abdominal segment.

**Figures 41–44.**
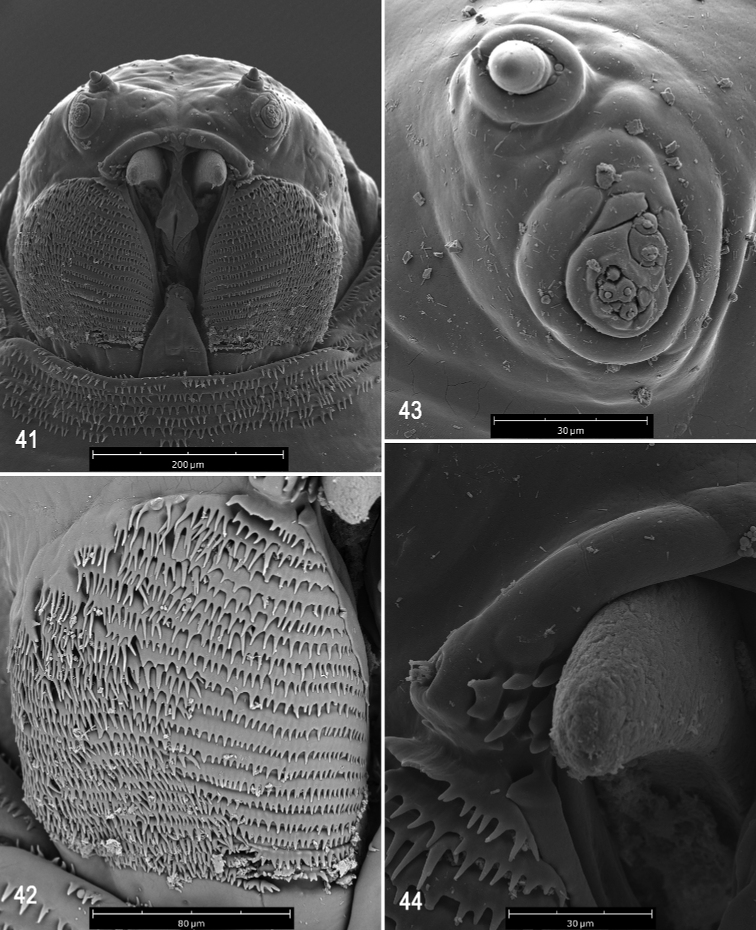
Scanning electron photomicrographs of third instar of *Anastrephahaplacantha***41** pseudocephalon **42** oral ridges **43** antenna and maxillary palp **44** preoral organ and ventral surface of mouthhook. Scale bars: 30 μm (**43, 44**); 80 μm (**42**); 200 μm (**41**).

***Pseudocephalon*** (Figs 41–44). Antenna and maxillary palp on moderately developed lobe. Antenna with cylindrical base and apical knob. Maxillary palp bearing three papilla sensilla, two knob sensilla; dorsolateral group of sensilla bearing two well-developed papilla sensilla, aligned at an oblique angle to palp and surrounded by collar. Facial mask globular in lateral view. Preoral organ bearing 2–4 peg sensilla, located apically on simple elongate preoral lobe lateral to mouthhook, 3–5 short elongate single or bifid secondary lobes adjacent to preoral organ. Oral ridges in 19 or 20 rows, posterior margins dentate with long moderately spaced projections; numerous accessory plates lateral to oral ridges, some elongate and interleaved with oral ridges, covering a much smaller area than oral ridges, with fringed posterior margins. Labium triangular, anterior surface with reclinate spines, ventrally with visible sensilla on small tubercles.

**Figures 45–50.**
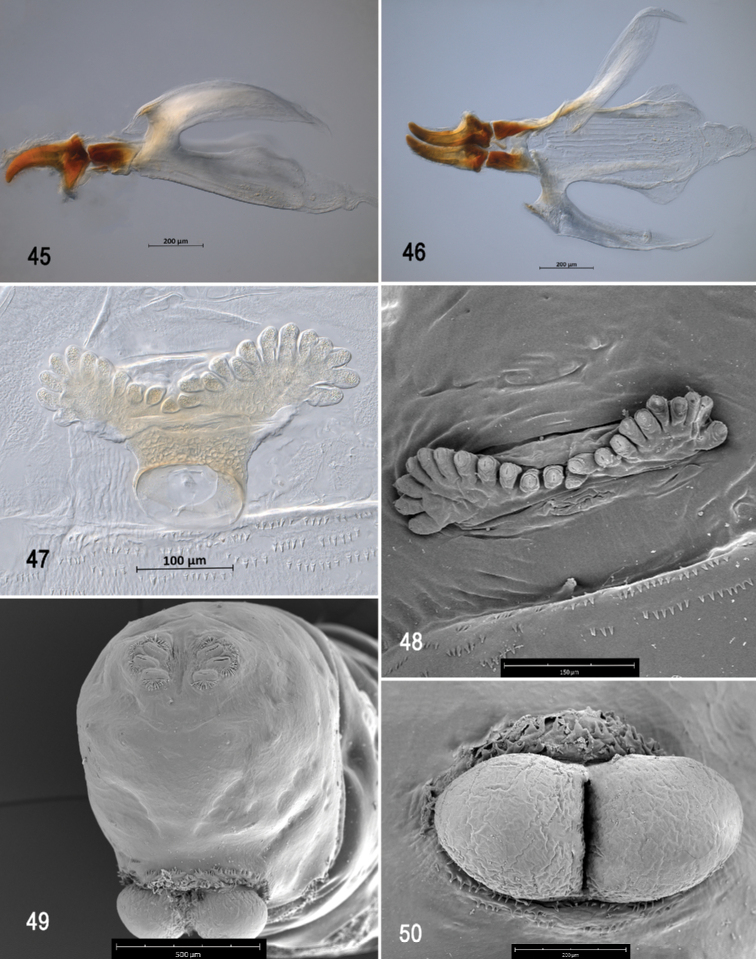
Optical photomicrographs and scanning electron photomicrographs of third instar of *Anastrephahaplacantha***45** cephaloskeleton, lateral view **46** cephaloskeleton, dorsal view **47** prothoracic spiracle, lateral view **48** prothoracic spiracle, dorsolateral view **49** caudal segment **50** anal lobe. Scale bars: 100 μm (**47**); 150 μm (**48**); 200 μm (**45, 46, 50**); 500 μm (**49**).

***Cephaloskeleton*** (Figs 45, 46). Total length from tip of mouthhook to end of ventral cornu 1.3 mm. Mouthhook well sclerotized, reddish orange; length a 0.31–0.32 mm; length b 0.21–0.22 mm; height c 0.22–0.24 mm; ratio a:b 1.45–1.46; ratio a:c 1.33–1.42. Tooth long, sharp, strongly curved, concave ventrally, ventral surface apparently smooth. Intermediate sclerite 0.20–0.23 mm long, 0.14 mm wide at ventral bridge. Epipharyngeal sclerite visible only in dorsal view, with medial lobe directed anteriorly. Labial sclerite robust, weakly sclerotized, and triangular in dorsal view. Parastomal bar extending three-fourths length of intermediate sclerite. Dorsal arch 0.25–0.26 mm high. Dorsal cornu weakly sclerotized, 0.49 mm long. Dorsal bridge prominently projecting anteriorly from dorsal cornu and sclerotized. Anterior sclerite absent. Cornu notch (N) 0.35 mm long and cornu notch index (N/DC) 0.7. Ventral cornu weakly sclerotized. Pharyngeal filter with weakly sclerotized anterior bar and 7–9 ridges forming a series of grooves along length of ventral cornu. Ventral cornu 0.85 mm long from pharyngeal bar to posterior end of grooves. Ventral cornu 1.7 × as long as sclerotized area of dorsal cornu.

***Thoracic and abdominal segments*.** Thoracic segments with dorsal spinules conical, symmetrical to slightly curved posteriorly; dorsal spinules pattern in rows as follows: T1 with 5‒7 rows, forming scalloped plates; T2 with three or four rows; T3 with one row; ventral spinule pattern as follows: T1 with seven rows; T2 with four rows; T3 with two rows. Abdominal segments (A1–A8) lacking dorsal spinules; ventral creeping welts present on all abdominal segments; ventral spinule pattern as follows: A1 with two or three rows; A2 with six rows; A3 with eight rows; A4 with eight or nine; A5 with eight or nine rows; A6 with seven or eight rows; A7 with eight rows; A8 with eight rows. Additional three rows of irregular spinules anterior and posterior to anal lobes, lateral rows apparently absent, spinules large, conical, distally sharp, pointing away from anal lobes.

***Prothoracic spiracle*** (Figs 47, 48). Bilobed, bearing 20–24 tubules, distally rounded and arranged in a single sinuous row. Spiracle distal width 0.32–0.35 mm; basal width 0.12‒0.13 mm at junction with trachea.

***Caudal segment*** (Figs 49, 50). Dorsal (D1 and D2), intermediate (I1 and I2), lateral (L1), and ventral (V1 and V2) tubercles and sensilla weakly developed; D1 distinctly anterior to D2. Intermediate tubercles I1 and I2 and associated sensilla weakly developed; I1 dorsal to I2. L1, V1 and V2 tubercles, and associated sensilla weakly developed. Anal lobe entire and protuberant.

**Figures 51–52.**
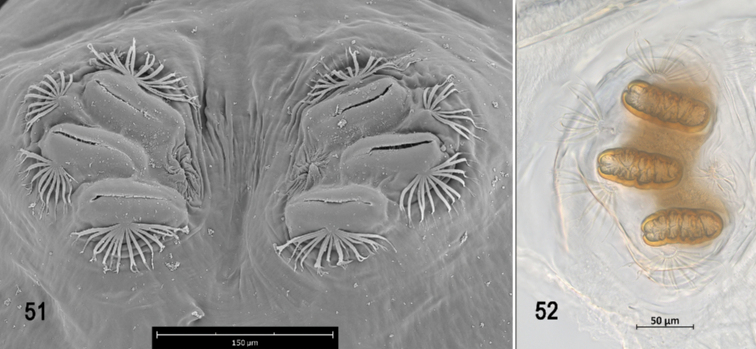
Scanning electron photomicrograph and optical photomicrograph of posterior spiracles of third instar of *Anastrephahaplacantha*. Scale bars: 50 μm (**52**); 150 μm (**51**).

***Posterior spiracle*** (Figs 49, 51, 52). Located above horizontal midline. Posterior spiracle openings with thick rimae and numerous trabeculae; 69–80 µm long; 27‒33 µm wide; ratio length/width 2.2‒2.8. Ecdysial scar apparent. Felt chamber oval, 158‒180 µm in diameter at junction with trachea. Spiracular process SP-I comprising 9‒12 trunks and 13‒27 tips; ratio tips/trunks 1.4‒ 2.3; basal width 12‒18 µm; ratio basal width/length of spiracular opening 0.16‒0.23. SP-II comprising 6‒9 trunks and 8‒19 tips. SP-III comprising 6‒11 trunks and 12‒24 tips. SP-IV comprising 9‒12 trunks and 16‒23 tips; ratio tips/trunks 1.8‒1.9; basal width 14‒15 µm; ratio basal width/length of spiracular opening 0.19‒0.21.

##### Distribution.

*Anastrephahaplacantha* is known only from Ecuador (Orellana) (Norrbom and Korytkowski 2012).

##### Biology.

We reared this species from fruit of *Quararibeamalacocalyx*, the first host plant record for *A.haplacantha*. The larvae feed only on the endocarp (developing seed) of the fruit.

##### Molecular identification.

COI barcodes were generated from three larvae and four adults and submitted to GenBank (MT654690, MT763901–MT763904, MT763941, MT763944). These data further confirm the identity of the described larvae. K2P distances among *A.haplacantha* ranged from 0.0–2.7%. BLAST searches were consistent with our new data, yielding only one good match: *A.haplacantha* (97% sequence identity; KY428381). Additionally, all three larval barcodes returned consensus identifications of *A.haplacantha* with three votes (Moore et al. in press).

#### ﻿ Anastrepha korytkowskii

Taxon classificationAnimaliaDipteraTephritidae

Norrbom, 2015

2322DD6E-FED3-58AF-8BAE-458A6E143CDA

Figs 53–57 , 58–63 , 64–65

##### Material examined.

Peru • 2 larvae; Madre de Dios, Puerto Maldonado, Centro de Investigación y Capacitación Río Los Amigos (CICRA), trail 21; 12.5721°S, 70.0889°W; 232 m a.s.l.; 17 Apr. 2016; N. Zenteno leg.; reared from fruit of *Quararibeawittii*; FSCA (AP20180315.02– AP20180315.03) • 7 larvae; same, trail 11; 12.5636°S, 70.0847°W; 250 m a.s.l.; 4 Dec. 2015; R. Bustamante leg.; reared from fruit of *Quararibeawittii*; FSCA (AP20180315.01, AP20180315.04, AP20180315.05, AP20180321.03, AP20180321.04, AP20180329.01, AP20180329.05) • 2 larvae; same, trail 21; 12.5708°S, 70.0847°W; 224 m a.s.l.; 2 Dec. 2015; R. Bustamante leg.; reared from fruit of *Quararibeawittii*; FSCA (AP20180315.07, AP20180516.13) • 8 larvae; same, trail 21; 12.5721°S, 70.0889°W; 232 m a.s.l.; 14–21 Mar. 2016; N. Zenteno leg.; reared from fruit of *Quararibeawittii*; FSCA (AP20180315.08, AP20180315.09, AP20180329.06, AP20180329.07, AP20180329.09–AP20180329.12).

**Figures 53–57.**
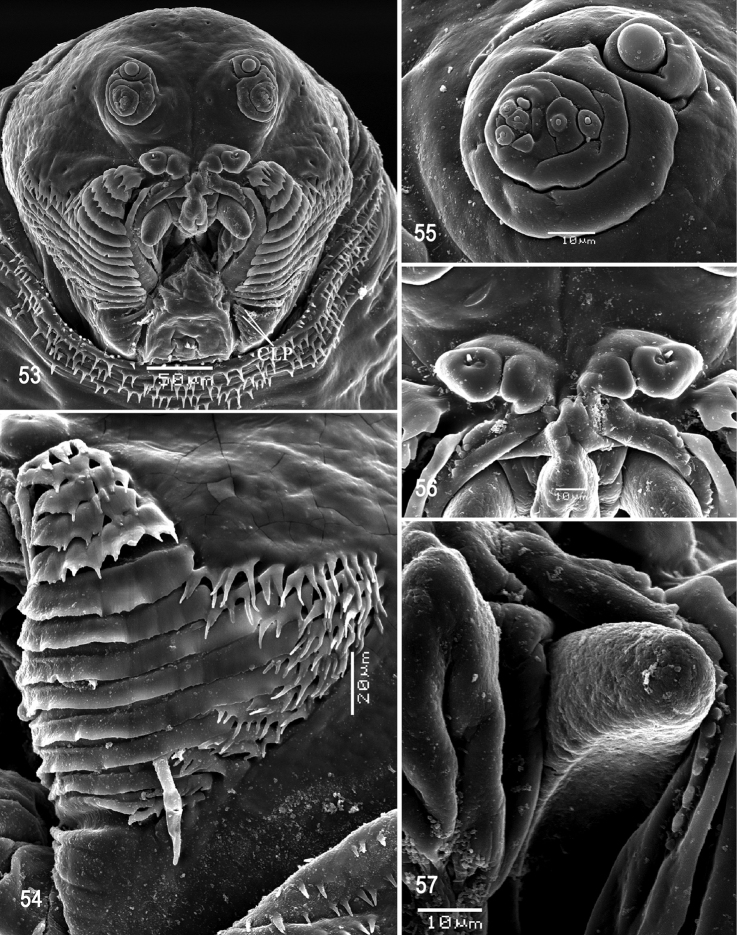
Scanning electron photomicrographs of third instar of *Anastrephakorytkowskii***53** pseudocephalon **54** oral ridges **55** antenna and maxillary palp **56** preoral organ **57** ventral surface of mouthhook. Abbreviations: CLP, comb-like processes. Scale bars: 10 μm (**55–57**); 20 μm (**54**); 50 μm (**53**).

##### Diagnosis.

The larvae of *A.korytkowskii* can be distinguished from those of other species of *Anastrepha* by its peculiar short preoral lobe medial to the lobe bearing the preoral organ, fringed posterior margins of the accessory plates, posterior margins of the oral ridges (2–5 anterior ridges dentate, medial and posterior ridges entire), and 3–5 comb-like processes adjacent to the labium and posterior to the oral ridges. The posterior margins of the accessory plates resemble those of *A.crebra*, *A.haplacantha*, *A.nolazcoae*, *Anastrepha* sp. Peru-82, Anastrephasp.nr.protuberans, and *Anastrepha* sp. Sur-16, although in *A.korytkowskii* the posterior margins of the oral ridges are distinct (as shown above). *Anastrephakorytkowskii* further differs from the latter six species by the number of oral ridges, ventral surface of mouthhook, number of tubules and distal width of the prothoracic spiracle, and basal width of the posterior spiracle (see Tables 2–4).

##### Description.

***Habitus*.** Third instar elongate, cylindrical, tapered anteriorly and caudal end truncate; color creamy; amphipneustic. Length 6.10‒8.54 mm and width 0.93‒1.57 mm at the sixth abdominal segment.

***Pseudocephalon*** (Figs 53–56). Antenna and maxillary palp on moderately developed lobe. Antenna with cylindrical base and apical knob. Maxillary palp bearing three papilla sensilla, two knob sensilla; dorsolateral group of sensilla bearing two well-developed papilla sensilla, aligned perpendicular to palp and surrounded by a collar. Facial mask partly globular in lateral view, upper right section lacking ridges and accessory plates and forming almost a right angle. Preoral organ bearing one unbranched peg sensillum, located apically on small, elongate-rounded lobe directly anterior to mouthhook; adjacent medial preoral lobe separate, slightly smaller and irregularly rounded. Oral ridges in 12–14 rows, margins of anterior 2–5 ridges irregularly dentate, margins of medial and posterior ridges entire (some sparsely notched); 3–5 comb-like processes adjacent to labium and posterior to oral ridges; 14–20 accessory plates in one series, but absent adjacent to the anterior five or six oral ridges, covering a much smaller area than oral ridges, with fringed posterior margins. Labium triangular, anterior surface knobby, ventrally with two visible sensilla.

**Figures 58–63.**
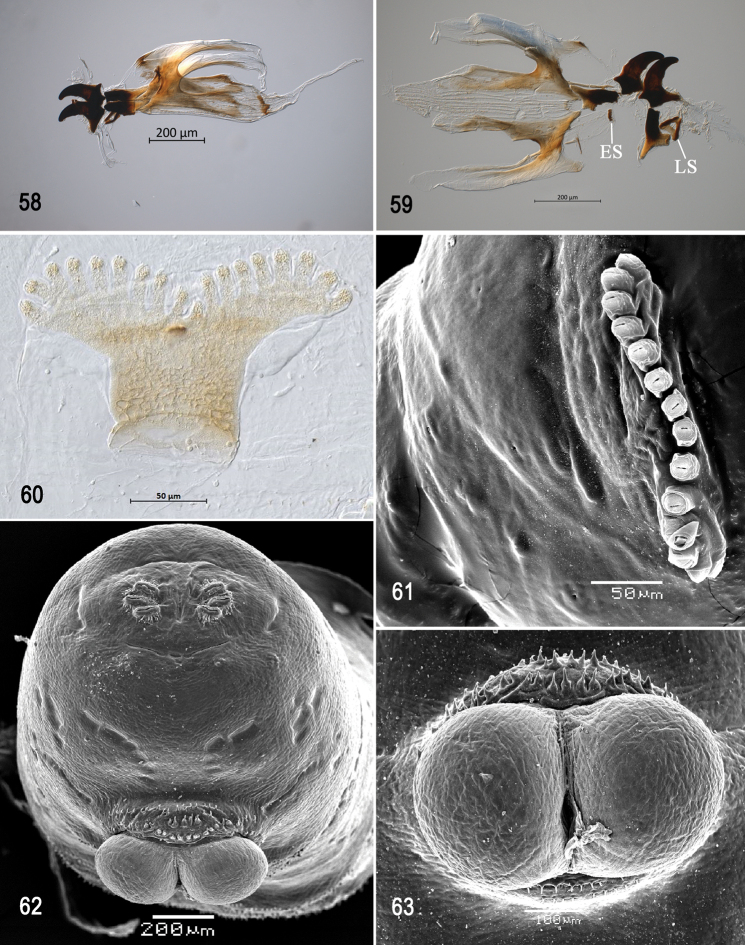
Optical photomicrographs and scanning electron photomicrographs of third instar of *Anastrephakorytkowskii***58** cephaloskeleton, lateral view **59** cephaloskeleton, dorsal view **60** prothoracic spiracle, lateral view **61** prothoracic spiracle, dorsolateral view **62** caudal segment **63** anal lobe. Abbreviations: ES, epipharyngeal sclerite; LS, labial sclerite. Scale bars: 50 μm (**60, 61**); 100 μm (**63**); 200 μm (**58, 59, 62**).

***Cephaloskeleton*** (Figs 57–59). Total length from tip of mouthhook to end of ventral cornu 0.76–0.86 mm. Mouthhook well sclerotized, black apically and basally; length a 0.16–0.18 mm; length b 0.10–0.13 mm; height c 0.11–0.13 mm; ratio a:b 1.4–1.6; ratio a:c 1.4–1.5. Tooth long, sharp, strongly curved, concave ventrally, ventral surface eroded. Intermediate sclerite 0.15–0.17 mm long, 0.13–0.14 mm wide at ventral bridge. Epipharyngeal sclerite visible only in dorsal view, with medial lobe directed anteriorly. Labial sclerite robust, sclerotized, and triangular in dorsal view. Parastomal bar extending for almost entire length of intermediate sclerite. Dorsal arch 0.19–0.21 mm high. Dorsal cornu with well-defined sclerotized area adjacent to notch, 0.36–0.46 mm long. Dorsal bridge prominently projecting anteriorly from dorsal cornu and slightly sclerotized. Anterior sclerite irregularly shaped and sclerotized. Cornu notch (N) 0.24–0.29 mm long and cornu notch index (N/DC) 0.6–0.7. Ventral cornu sclerotized between notch and pharyngeal bar and grooves. Pharyngeal filter with weakly sclerotized anterior bar and 7–9 ridges forming a series of grooves along length of ventral cornu. Ventral cornu 0.39–0.55 mm long from pharyngeal bar to posterior end of grooves. Ventral cornu 1.2–1.4 × as long as sclerotized area of dorsal cornu.

***Thoracic and abdominal segments*.** Thoracic segments with dorsal spinules conical, symmetrical to slightly curved posteriorly; dorsal spinule pattern as follows: T1 with six or seven rows, forming scalloped plates; T2 with 2‒5 rows; T3 lacking spinules; ventral spinule pattern as follows: T1 with 8–12 rows; T2 with three rows; T3 lacking spinules. Abdominal segments (A1–A8) lacking dorsal spinules; ventral creeping welts present on all abdominal segments (A1–A8); ventral spinule pattern as follows: A1 with one or two rows; A2 with six or seven rows; A3 with seven or eight rows; A4 with seven or eight rows; A5 with 6–8 rows; A6 with eight rows; A7 with 6–8 rows; A8 with 6–8 rows. Additional 2–4 irregular rows of spinules anteriorly and posteriorly to anal lobes, spinules large, conical, pointing away from anal lobes.

**Figures 64, 65.**
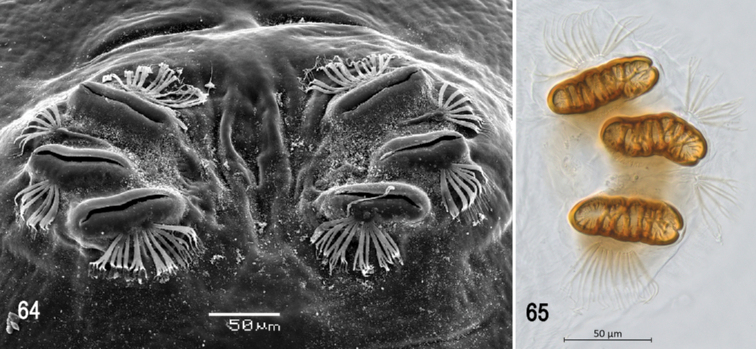
Scanning electron photomicrograph and optical photomicrograph of posterior spiracles of third instar of *Anastrephakorytkowskii*. Scale bars: 50 μm (**64, 65**).

***Prothoracic spiracle*** (Figs 60, 61). Bilobed, bearing 12–18 tubules, distally rounded and arranged in a single sinuous row. Spiracle distal width 0.19–0.24 mm; basal width 0.07‒0.10 mm at junction with trachea.

***Caudal segment*** (Figs 62, 63). Dorsal (D1 and D2), intermediate (I1 and I2), lateral (L1), and ventral (V1 and V2) tubercles and sensilla weakly developed; D1 distinctly anterior to D2. Intermediate tubercles I1 and I2 more strongly developed, but associated sensilla weakly developed; I1 lateral and sometimes slightly ventral to I2. Lateral (L1) and ventral (V1 and V2) tubercles and associated sensilla weakly developed. Anal lobe entire and very protuberant.

***Posterior spiracle*** (Figs 62, 64, 65). Located above horizontal midline. Posterior spiracle openings with thick rimae and numerous trabeculae; 56–77 µm long; 20‒24 µm wide; ratio length/width 2.8‒3.2. Ecdysial scar apparent. Felt chamber oval, 124‒148 µm in diameter at junction with trachea. Spiracular process SP-I comprising 9‒15 trunks and 21‒33 tips; ratio tips/trunks 2.2‒ 2.3; basal width 14‒28 µm; ratio basal width/length of spiracular opening 0.24‒0.47. SP-II comprising 4‒7 trunks and 9‒17 tips. SP-III comprising 5‒10 trunks and 12‒19 tips. SP-IV comprising 8‒15 trunks and 17‒31 tips; ratio tips/trunks 2.0‒2.1; basal width 12‒21 µm; ratio basal width/length of spiracular opening 0.21‒0.30.

##### Distribution.

*Anastrephakorytkowskii* is known only from Bolivia (La Paz and Santa Cruz) and eastern Peru (Cusco, Huánuco, Junín, and Madre de Dios).

##### Biology.

We reared this species from fruit of *Quararibeawittii*, the only known host plant (Norrbom et al. 2015). The larvae feed only on the pulp of the fruit.

##### Molecular identification.

COI barcodes were generated from 19 larvae and two adults and submitted to GenBank (MT654705–MT654725). These data further confirm the identity of the described larvae. K2P distances between *A.korytkowskii* larvae and the three adult sequences (MT654712, MT654722, KY428387) ranged from 0.0–2.1%. BLAST searches were consistent with our new data, yielding only one good match: *A.korytkowskii* (97.77–99.04% sequence identity; KY428387). Additionally, all 19 larval barcodes returned consensus identifications of *A.korytkowskii* with three votes (Moore et al. in press).

#### ﻿ Anastrepha nolazcoae

Taxon classificationAnimaliaDipteraTephritidae

Norrbom & Korytkowski, 2011

A7DD3B4D-0B13-5BDD-836A-42A92DCA9644

Figs 66–69 , 70–75 , 76–80

##### Material examined.

Peru • 20 larvae; Madre de Dios, Puerto Maldonado, Centro de Investigación y Capacitación Río Los Amigos (CICRA), trail 21; 12.5722°S, 70.0885°W; 233 m a.s.l.; 1–5 Feb. 2014; E. J. Rodriguez and J. Caballero leg.; reared from fruit of *Quararibeacordata*; FSCA (AP20180222.01–AP20180222.10, AP20180206.01–AP20180206.10).

##### Diagnosis.

The larva of *A.nolazcoae* differs from those of all other species of *Anastrepha* that have been adequately described by the combination of having fringed posterior margins of the oral ridges and accessory plates, and the presence of 6–8 comb-like processes adjacent to the labium. The posterior margins of the oral ridges and accessory plates resemble those of *A.crebra*, *A.haplacantha*, *Anastrepha* sp. Peru-82, and Anastrephasp.nr.protuberans, but those species lack the comb-like processes. In addition, *A.nolazcoae* resembles *A.korytkowskii* and *Anastrepha* sp. Sur-16 in the presence of comb-like processes, but *A.nolazcoae* can be distinguished from them by the fringed posterior margins of its oral ridges. Other characters such as the ventral surface of the mouthhook, number of tubules and apical width of the prothoracic spiracle, and dorsal spinules on thoracic segments further differentiate *A.nolazcoae* (see Tables 2–4).

*Anastrephanolazcoae* shares the same host plant, *Quararibeacordata*, with species within the *fraterculus* group (*A.fraterculus* complex), *mucronota* group (*A.mucronota*), and *striata* group (*A.striata*). The larva of *A.mucronota* was described with limited data (Steyskal 1977) but can be morphologically separated from *A.nolazcoae* by the lower number of oral ridges (13–15 vs. 16–19) and dorsal irregularly light brown plaques on the abdominal segments (present vs. absent). The description of *A.mucronota* lacks information for most of the characters of the pseudocepalon (Table 2) and most of the available data overlap with those of *A.nolazcoae* (Table 3, 4). *Anastrephanolazcoae* differs from five morphotypes within the *A.fraterculus* complex (Canal et al. 2015, 2018) and *A.striata* as follows: 1) greater number of oral ridges (16–19; see the dichotomous key in Steck et al. 1990), except unknown for Andean and Peruvian morphotypes of *A.fraterculus* complex; 2) posterior margin of oral ridges fringed in *A.nolazcoae*, irregularly serrate in *A.fraterculus* (Brazil-1 and Ecuadorian morphotypes), scalloped or emarginate in *A.fraterculus* (Mexican morphotype), entire or serrate in *A.striata*; and 3) approximately 36 accessory plates with fringed posterior margins in *A.nolazcoae*, apparently seven plates and serrate in *A.fraterculus* (Ecuadorian morphotypes; see plate 4b in White and Elson-Harris 1992), eight plates and serrate in *A.fraterculus* (Mexican morphotype), 8–9 plates and entire in *A.striata*. *Anastrephanolazcoae* differs further from the *A.fraterculus* complex in having a greater number of tubules on the prothoracic spiracle (18–21 vs. 9–18 in *fraterculus* complex, see Rodriguez et al. 2021), although in this character it overlaps with *A.striata*.

**Figures 66–69.**
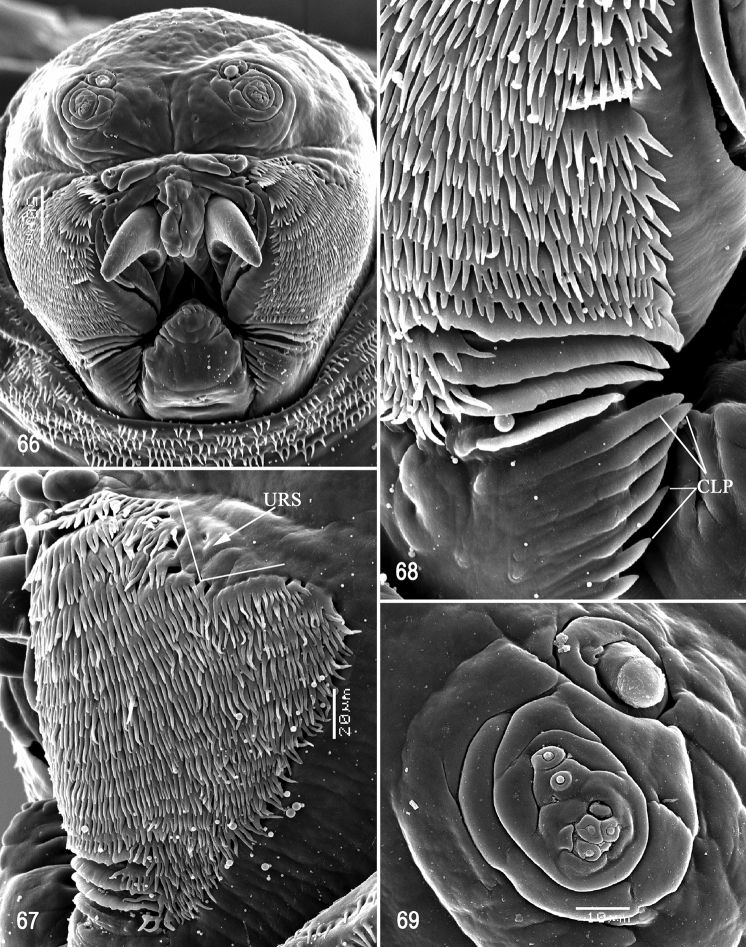
Scanning electron photomicrographs of third instar of *Anastrephanolazcoae***66** pseudocephalon **67** oral ridges **68** comb-like processes **69** antenna and maxillary palp. Abbreviations: CLP, comb-like processes; URS, upper right section with right angle shape. Scale bars: 10 μm (**69**); 20 μm (**67**); 50 μm (**66**).

##### Description.

***Habitus*.** Third instar elongate, cylindrical, tapered anteriorly and caudal end truncate; color creamy; amphipneustic. Length 5.33‒11.76 mm and width 0.93‒1.92 mm at the sixth abdominal segment.

**Figures 70–75.**
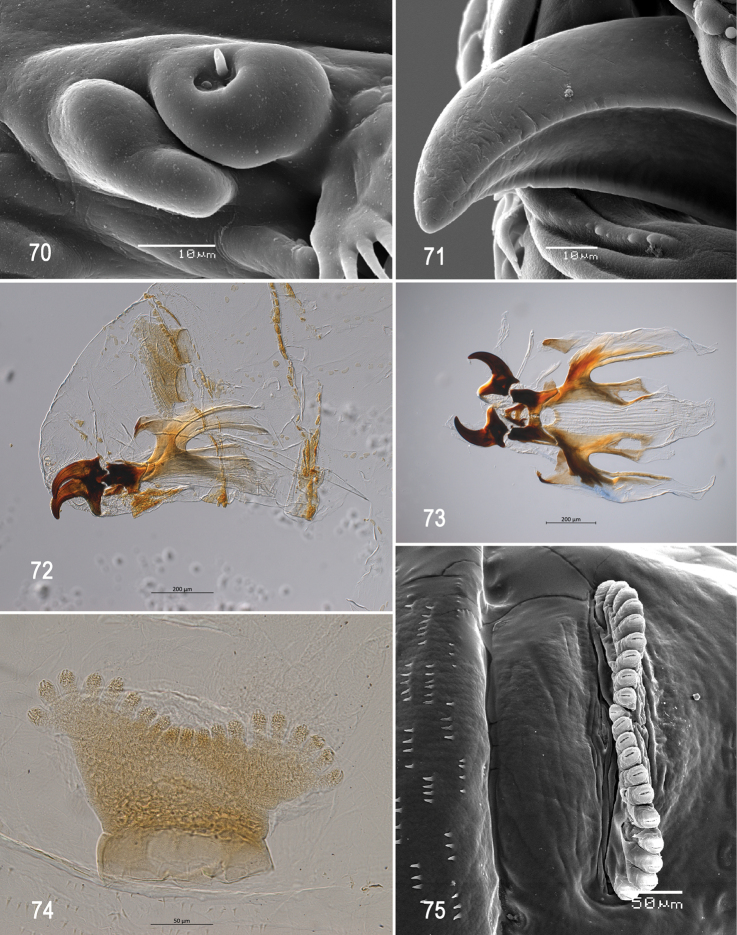
Optical photomicrographs and scanning electron photomicrographs of third instar of *Anastrephanolazcoae***70** preoral organ **71** ventral surface of mouthhook **72** cephaloskeleton, lateral view **73** cephaloskeleton, dorsal view **74** prothoracic spiracle, lateral view **75** prothoracic spiracle, dorsolateral view. Scale bars: 10 μm (**70, 71**); 50 μm (**74, 75**); 200 μm (**72, 73**).

***Pseudocephalon*** (Figs 66–70). Antenna and maxillary palp on moderately developed lobe. Antenna with cylindrical base and apical knob. Maxillary palp bearing three papilla sensilla, two knob sensilla; dorsolateral group of sensilla bearing two well-developed papilla sensilla, aligned perpendicular to palp and surrounded by collar. Facial mask partly globular in lateral view, upper right section lacking ridges and accessory plates and forming almost a right angle. Preoral organ bearing one unbranched peg sensillum, located apically on a small, elongate-rounded lobe directly anterior to mouthhook; adjacent medial preoral lobe separate, short-elongate, extending partially posterior to lobe bearing preoral organ. Oral ridges in 16–19 rows, 13–15 anterior ridges with fringed posterior margins, three or four posterior ridges entire, undulant; 6–8 comb-like processes adjacent to labium and posterior to oral ridges; approximately 36 accessory plates lateral to oral ridges covering a much smaller area than oral ridges, with fringed posterior margins as on oral ridges, in two series. Labium triangular, anterior surface knobby, ventrally with two visible sensilla.

**Figures 76–80.**
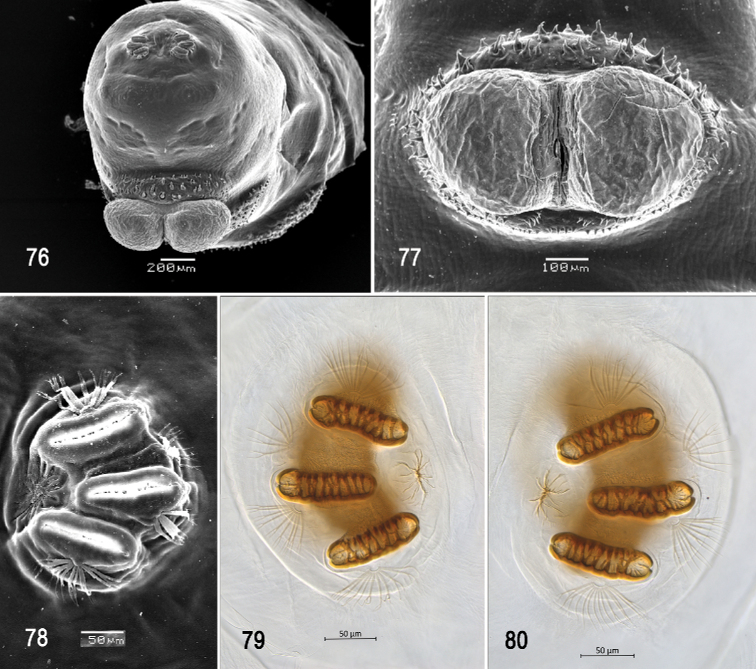
Scanning electron photomicrographs and optical photomicrographs of third instar of *Anastrephanolazcoae***76** caudal segment **77** anal lobe **78–80** posterior spiracles. Scale bars: 50 μm (**78–80**); 100 μm (**77**); 200 μm (**76**).

***Cephaloskeleton*** (Figs 71–73). Total length from tip of mouthhook to end of ventral cornu 0.69–1.10 mm. Mouthhook well sclerotized, black apically and basally; length a 0.20–0.23 mm; length b 0.12–0.15 mm; height c 0.14–0.17 mm; ratio a:b 1.5–1.7; ratio a:c 1.3–1.4. Tooth long, sharp, strongly curved, concave ventrally with weak medial carina, ventral surface smooth. Intermediate sclerite 0.16–0.20 mm long, 0.18–0.21 mm wide at ventral bridge. Epipharyngeal sclerite visible only in dorsal view, with medial lobe directed anteriorly. Labial sclerite robust, sclerotized, and triangular in dorsal view. Parastomal bar extending three-fourths length of intermediate sclerite. Dorsal arch 0.23–0.29 mm high. Dorsal cornu with well-defined sclerotized area adjacent to notch, 0.38–0.53 mm long. Dorsal bridge prominently projecting anteriorly from dorsal cornu and slightly sclerotized. Anterior sclerite irregularly shaped and sclerotized. Cornu notch (N) 0.25–0.34 mm long and cornu notch index (N/DC) 0.6–0.7. Ventral cornu with well-defined sclerotized area between notch and pharyngeal bar and grooves. Pharyngeal filter with weakly sclerotized anterior bar and seven ridges forming a series of grooves along length of ventral cornu. Ventral cornu 0.44–0.71 mm long from pharyngeal bar to posterior end of grooves. Ventral cornu 1.18–1.34 × as long as sclerotized area of dorsal cornu.

***Thoracic and abdominal segments*.** Thoracic segments with dorsal spinules conical, symmetrical to slightly curved posteriorly; dorsal spinule pattern as follows: T1 with 3‒5 rows; T2 with 3‒5 rows; T3 with one or two rows; ventral spinule pattern as follows: T1 with 8‒11 rows; T2 with four or five rows; T3 with three or four rows. Abdominal segments (A1–A8) lacking dorsal spinules; ventral creeping welts present on all abdominal segments (A1–A8); ventral spinule pattern as follows: A1 with three or four rows; A2 with six or seven rows; A3–A6 with 6–8 rows; A7 with six or seven rows; A8 with 6–9 rows. Additional two or three irregular rows of spinules anteriorly and posteriorly to anal lobes, two rows laterally, spinules large, conical, pointing away from anal lobes.

***Prothoracic spiracle*** (Figs 74, 75). Bilobed, bearing 18–21 tubules, distally rounded and arranged in a single sinuous row. Spiracle distal width 0.26–0.34 mm; basal width 0.12‒0.17 mm at junction with trachea.

***Caudal segment*** (Figs 76, 77). Dorsal (D1 and D2), intermediate (I1 and I2), lateral (L1), and ventral (V1 and V2) tubercles and sensilla weakly developed; D1 distinctly anterior to D2. Intermediate tubercles I1 and I2 more strongly developed, but associated sensilla weakly developed; I1 lateral and sometimes slightly ventral to I2. L1, V1, and V2 tubercles and associated sensilla weakly developed. Anal lobe entire and very protuberant.

***Posterior spiracle*** (Figs 76, 78–80). Located above horizontal midline. Posterior spiracle openings with thick rimae and numerous trabeculae; 83–108 µm long; 27‒32 µm wide; ratio length/width 3.0‒3.4. Ecdysial scar apparent. Felt chamber oval, 187‒210 µm in diameter at junction with trachea. Spiracular process SP-I comprising 8‒11 trunks and 9‒26 tips; ratio tips/trunks 1.1‒ 2.4; basal width 9‒15 µm; ratio basal width/length of spiracular opening 0.09‒0.17. SP-II comprising 3‒7 trunks and 6‒14 tips. SP-III comprising 3‒9 trunks and 5‒20 tips. SP-IV comprising 4‒12 trunks and 8‒24 tips; ratio tips/trunks 2.0; basal width 7‒12 µm; ratio basal width/length of spiracular opening 0.08‒0.12.

##### Distribution.

*Anastrephanolazcoae* is known only from Peru (Amazonas, Cajamarca, Huánuco, San Martín) (Norrbom and Korytkowski 2011; Barr et al. 2017; Bartolini et al. 2020).

##### Biology.

We reared this species from fruit of *Quararibeacordata*, the only known host. It was previously reared from the same fruit in Peru: Huánuco: Tingo Maria (Norrbom and Korytkowski 2011). The larvae feed only on the pulp of the fruit.

##### Molecular identification.

COI barcodes were generated for 29 larvae and five adults and submitted to GenBank (MH070234, MT643950–MT643954, MT654802–MT654827, MT884299, MT884396). These data further confirm the identity of the described larvae. K2P distances between *A.nolazcoae* larvae and the nine available adult sequences ranged from 0.0–1.1%. BLAST searches were consistent with our new data, yielding only four good matches: *A.nolazcoae* (99.21–100% sequence identity; KY428297, MN454445, MN454488, MF695205 [identified as *A.kuhlmanni* in GenBank, reported as *A.nolazcoae* in Barr et al. 2017]). Additionally, 27 larval barcodes returned consensus identifications of *A.nolazcoae* with either three or two votes, and two samples returned ambiguous identifications (Moore et al. in press).

#### ﻿ Anastrepha

Taxon classificationAnimaliaDipteraTephritidae

sp. Peru-82

C61F1AED-2EC7-50FC-9CBA-F73DD8755A73

Figs 81–85 , 86–91 , 92–94

##### Material examined.

Peru • 6 larvae; Loreto, Iquitos, ExplorNapo, main trail; 3.2547°S, 72.9133°W; 132 m a.s.l.; 11 Feb. 2015; E. J. Rodriguez and J. Caballero leg.; reared from fruit of *Scleronemapraecox*; FSCA (AP20180109.02, AP20180124.03, AP20180124.04, AP20190827.10– AP20190827.12).

##### Diagnosis.

The larva of *Anastrepha* sp. Peru-82 differs from those of other species of *Anastrepha*, except *A.crebra*, *A.haplacantha*, *A.korytkowskii*, *A.nolazcoae*, Anastrephasp.nr.protuberans, and *Anastrepha* sp. Sur-16, in having the posterior margins of the accessory plates fringed. It differs from all other species except *A.korytkowskii*, *A.nolazcoae*, and *Anastrepha* sp. Sur-16 by the position of its preoral organ anterior to the mouthhook, and short preoral lobe. *Anastrepha* sp. Peru-82 can be further distinguished from *A.crebra* in having a higher number of oral ridges, and it further differs from *A.korytkowskii*, *A.nolazcoae*, and *Anastrepha* sp. Sur-16 in lacking comb-like processes adjacent to the labium. The number of tubules on the prothoracic spiracle and the dorsal spinule pattern on the thoracic segments are useful to further distinguish *Anastrepha* sp. Peru-82 from other species in the *mucronota* group (see Table 3).

##### Description.

***Habitus*.** Third instar elongate, cylindrical, tapered anteriorly and caudal end truncate; color creamy; amphipneustic. Length 8.71‒10.94 mm and width 1.40‒1.72 mm at the sixth abdominal segment.

**Figures 81–85.**
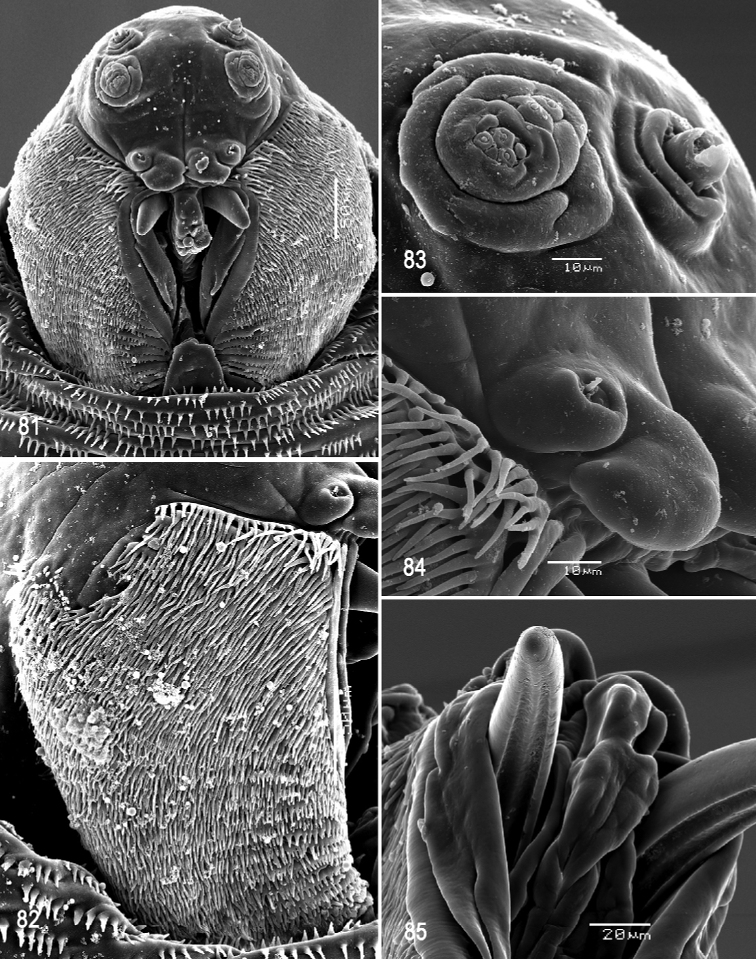
Scanning electron photomicrographs of third instar of *Anastrepha* sp. Peru-82 **81** pseudocephalon **82** oral ridges **83** antenna and maxillary palp **84** preoral organ **85** ventral surface of mouthhook. Scale bars: 10 μm (**83, 84**); 20 μm (**85**); 50 μm (**81, 82**).

***Pseudocephalon*** (Figs 81–84). Antenna and maxillary palp on moderately developed lobe. Antenna with cylindrical base and apical knob. Maxillary palp bearing three papilla sensilla, two knob sensilla; dorsolateral group of sensilla bearing two well-developed papilla sensilla, aligned perpendicular to palp and surrounded by collar. Facial mask partly globular in lateral view, upper right section lacking ridges and accessory plates and forming almost a right angle. Preoral organ bearing one unbranched peg sensillum, located apically on a small, rounded lobe directly anterior to mouthhook; adjacent medial preoral lobe of broad, irregular shape, approximately double size of lobe bearing preoral organ and extending partially posterior to it. Oral ridges in 22 or 23 rows, all densely fringed with very long, thin, tapering, pointed projections, but 8–12 posterior ridges with short weakly dentate section medially; numerous accessory plates present, with fringed posterior margins, in one or more series and overlapping with oral ridges (unable to distinguish end points). Labium triangular, anterior surface knobby (not clearly visible in Fig. 81), ventrally with visible sensilla.

**Figures 86–91.**
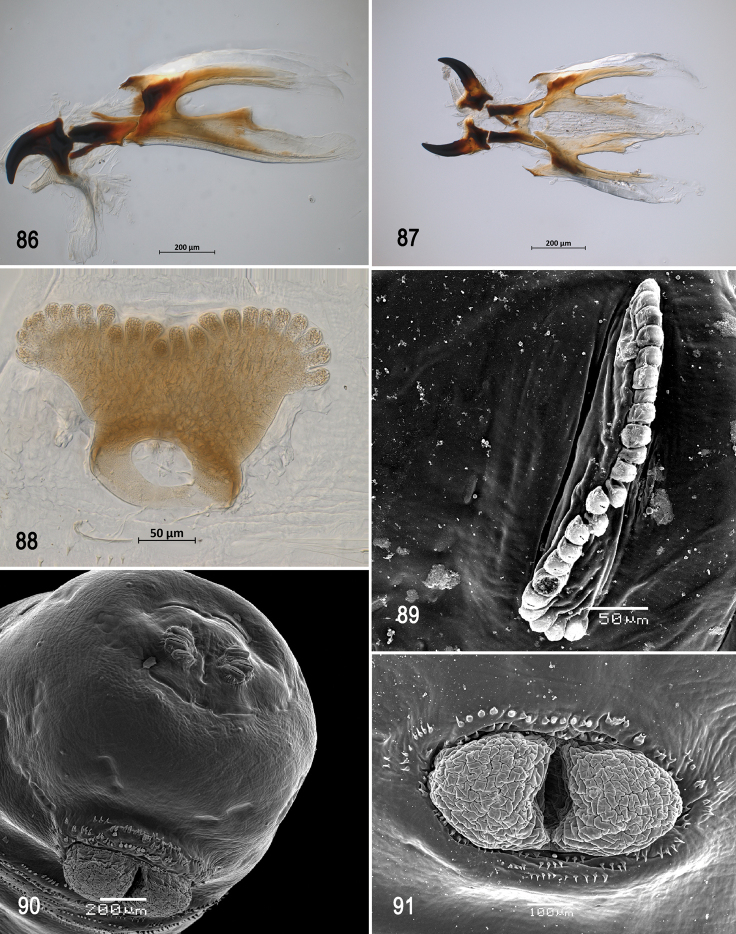
Optical photomicrographs and scanning electron photomicrographs of third instar of *Anastrepha* sp. Peru-82 **86** cephaloskeleton, lateral view **87** cephaloskeleton, dorsal view **88** prothoracic spiracle, lateral **89** prothoracic spiracle, dorsolateral **90** caudal segment **91** anal lobe. Scale bars: 50 μm (**88, 89**); 100 μm (**91**); 200 μm (**86, 87, 90**).

***Cephaloskeleton*** (Figs 85–87). Total length from tip of mouthhook to end of ventral cornu 1.0–1.28 mm. Mouthhook well sclerotized, black apically and basally; length a 0.25–0.28 mm; length b 0.18–0.20 mm; height c 0.17–0.20 mm; ratio a:b 1.31–1.41; ratio a:c 1.39–1.50. Tooth long, sharp, strongly curved, concave ventrally with medial carina and smooth surface. Intermediate sclerite 0.23–0.26 mm long, 0.14 mm wide at ventral bridge. Epipharyngeal sclerite visible only in dorsal view, with medial lobe directed anteriorly. Labial sclerite robust, sclerotized, and triangular in dorsal view. Parastomal bar extending three-fourths length of intermediate sclerite. Dorsal arch 0.22–0.24 mm high. Dorsal cornu with well-defined sclerotized area adjacent to notch, 0.48–0.64 mm long. Dorsal bridge prominently projecting anteriorly from dorsal cornu and strongly sclerotized. Anterior sclerite irregularly shaped and sclerotized. Cornu notch (N) 0.30–0.43 mm long and cornu notch index (N/DC) 0.63–0.67. Ventral cornu with well-defined sclerotized area from notch to pharyngeal bar and grooves. Pharyngeal filter with weakly sclerotized anterior bar and seven ridges forming a series of grooves along length of ventral cornu. Ventral cornu 0.58–0.81 mm long from pharyngeal bar to posterior end of grooves. Ventral cornu 1.20–1.45 × as long as sclerotized area of dorsal cornu.

**Figures 92–94.**
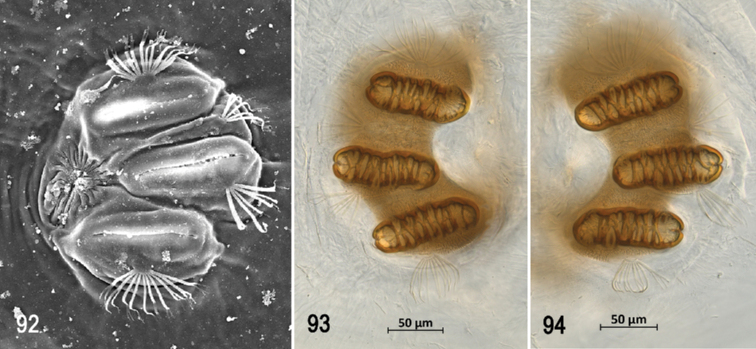
Scanning electron photomicrograph and optical photomicrographs of posterior spiracles of third instar of *Anastrepha* sp. Peru-82. Scale bars: 50 μm (**93, 94**).

***Thoracic and abdominal segments*.** Thoracic segments with dorsal spinules conical, symmetrical to slightly curved posteriorly; dorsal spinule pattern as follows: T1 with two rows; T2 with five or six rows; T3 with two or three rows; ventral spinules as follows: T1 with 7‒10 rows; T2 with 3–5 rows; T3 with two or three rows. Abdominal segments (A1–A8) lacking dorsal spinules, except A1 with three rows; ventral creeping welts present on all abdominal segments; ventral spinule pattern as follows: A1 with three or four rows; A2 with 7–9 rows; A3 with eight or nine rows; A4 with nine or ten rows; A5 with ten rows; A6 with 8–10 rows; A7 with 9–11 rows; A8 with 6–9 rows. Additional three irregular rows of spinules anteriorly and posteriorly to anal lobes, two rows laterally; spinules large, conical, pointing away from anal lobes.

***Prothoracic spiracle*** (Figs 88, 89). Bilobed, bearing 23–29 tubules, distally rounded and arranged in a single sinuous row. Spiracle distal width 0.28–0.35 mm; basal width 0.12‒0.16 mm at junction with trachea.

***Caudal segment*** (Figs 90, 91). Dorsal (D1 and D2), intermediate (I1 and I2), lateral (L1), and ventral (V1 and V2) tubercles and sensilla weakly developed; D1 distinctly anterior to D2. Intermediate tubercles I1 and I2 more strongly developed, but associated sensilla weakly developed; I1 lateral and sometimes slightly ventral to I2. L1, V1 and V2 most very weakly developed. Anal lobe entire and moderately protuberant.

***Posterior spiracle*** (Figs 90, 92–94). Located above horizontal midline. Posterior spiracle openings with thick rimae and numerous trabeculae; 84–97 µm long; 29‒34 µm wide; ratio length/width 2.6‒3.0. Ecdysial scar apparent. Felt chamber oval, 185‒212 µm in diameter at junction with trachea. Spiracular process SP-I comprising 9‒11 trunks and 12‒20 tips; ratio tips/trunks 1.3‒ 1.8; basal width 12‒15 µm; ratio basal width/length of spiracular opening 0.14‒0.16. SP-II comprising 4‒5 trunks and 5‒12 tips. SP-III comprising 4‒8 trunks and 5‒13 tips. SP-IV comprising 7‒11 trunks and 13‒16 tips; ratio tips/trunks 1.45‒1.85; basal width 9‒19 µm; ratio basal width/length of spiracular opening 0.11‒0.19.

##### Distribution.

*Anastrepha* sp. Peru-82 is only known from Peru (Loreto).

##### Biology.

We reared this species from fruit of *Scleronemapraecox*, the first host plant record. The larvae feed only on the pulp of the fruit.

##### Molecular identification.

COI barcodes were generated from six larvae and two adults and submitted to GenBank (MT644049–MT644051, MT763894–MT763898). These data further confirm the identity of the described larvae. K2P distances between *Anastrepha* sp. Peru-82 larvae and the adult sequences ranged from 0.0–1.1%. BLAST searches yielded no close matches to sequences from other *Anastrepha* species. Six larval barcodes returned consensus identifications of *Anastrepha* sp. Peru 82 with either three or two votes (Moore et al. in press).

#### ﻿ Anastrepha sp. near protuberans

Taxon classificationAnimaliaDipteraTephritidae

EAB6F498-A56B-5DEC-965F-2C24B9AEA425

Figs 95–99 , 100–105 , 106–108

##### Material examined.

Ecuador • 5 larvae; Orellana, Estacion Cientifica Yasuní, trail 6, near tower; 0.6805°S, 76.3851°W; 247 m a.s.l.; 6 Jan. 2018; M. R. Steck, G. J. Steck, E. J. Rodriguez and A. Padilla leg.; reared from fruit of *Sterculiafrondosa* Rich. (Malvaceae); FSCA (AP20180321.01, AP20180321.02, AP20200622.09–AP20200622.11).

##### Diagnosis.

The larva of Anastrepha sp. near protuberans differs from those of other species of *Anastrepha* except *A.crebra*, *A.haplacantha*, A.korytkowskii, *A.nolazcoae*, *Anastrepha* sp. Peru-82, and *Anastrepha* sp. Sur-16 by the fringed posterior margins of their oral ridges and accessory plates. Anastrepha sp. near protuberans can be distinguished from the latter six species in having a greater apical width of the prothoracic spiracle and slit length of the posterior spiracle. The number of oral ridges, number of tubules on the prothoracic spiracle, and dorsal spinule pattern on the thoracic segments further distinguish Anastrepha sp. near protuberans from species in the *mucronota* group (see Tables 2, 3).

##### Description.

***Habitus*.** Third instar elongate, cylindrical, tapered anteriorly and caudal end truncate; color creamy; amphipneustic. Length 14.43‒17.15 mm and width 2.52‒2.68 mm at the sixth abdominal segment.

**Figures 95–99.**
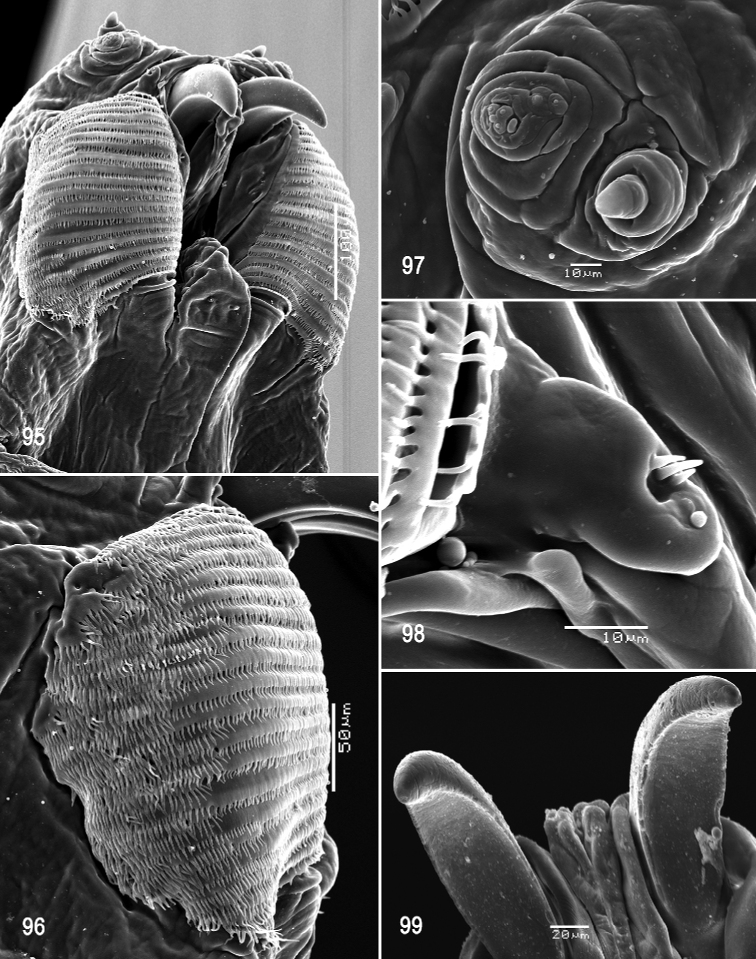
Scanning electron photomicrographs of third instar of Anastrephasp.nr.protuberans**95** pseudocephalon **96** oral ridges **97** antenna and maxillary palp **98** preoral organ **99** ventral surface of mouthhook. Scale bars: 10 μm (**97, 98**); 20 μm (**99**); 50 μm (**96**); 50 μm (**95**).

***Pseudocephalon*** (Figs 95–98). Antenna and maxillary palp on moderately developed lobe. Antenna with cylindrical base and apical knob. Maxillary palp bearing three papilla sensilla, two knob sensilla; dorsolateral group of sensilla bearing two well-developed papilla sensilla, aligned at strongly oblique angle to palp and surrounded by a collar. Facial mask globular in lateral view. Preoral organ bearing three unbranched peg sensilla, located apically on simple elongate preoral lobe lateral to mouthhook. Oral ridges in 18–23 rows, posterior margins densely and evenly fringed; accessory plates present covering a much smaller area than oral ridges, with fringed posterior margins longer than oral ridges, apparently in one series. Labium triangular, anterior surface knobby, ventrally with two visible sensilla and tubercles.

**Figures 100–105.**
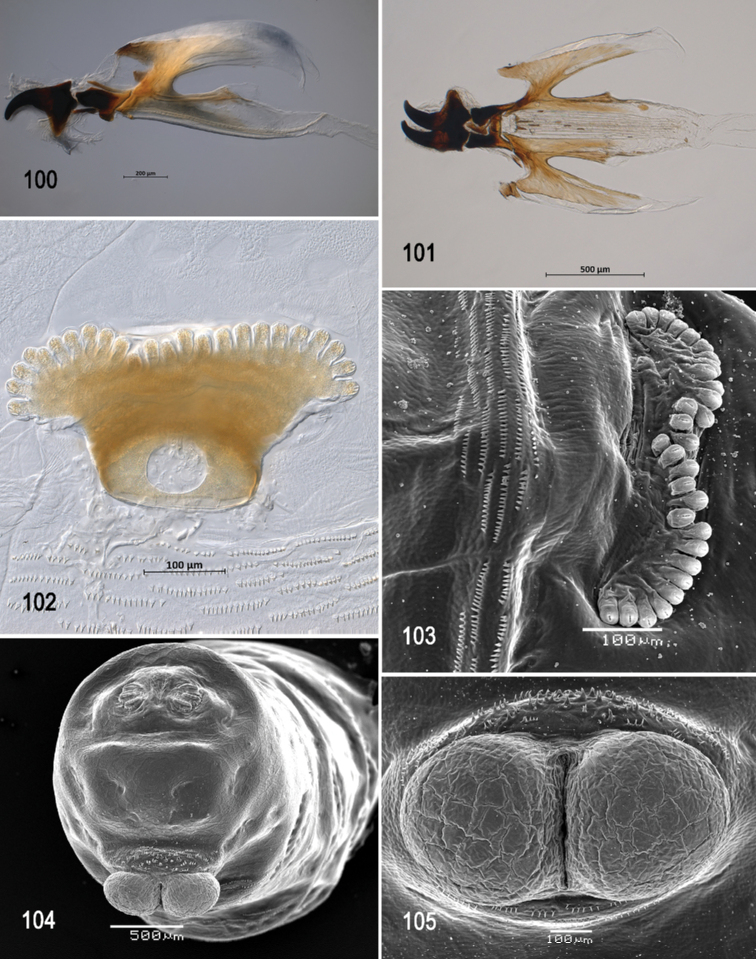
Optical photomicrographs and scanning electron photomicrographs of third instar of Anastrephasp.nr.protuberans**100** cephaloskeleton, lateral view **101** cephaloskeleton, dorsal view **102** prothoracic spiracle, lateral view **103** prothoracic spiracle, dorsolateral view **104** caudal segment **105** anal lobe. Scale bars: 100 μm (**102, 103, 105**); 200 μm (**100**); 500 μm (**101, 104**).

***Cephaloskeleton*** (Figs 99–101). Total length from tip of mouthhook to end of ventral cornu 1.48–1.51 mm. Mouthhook well sclerotized, black apically and basally; length a 0.34–0.35 mm; length b 0.24–0.25 mm; height c 0.26–0.28 mm; ratio a:b 1.41–1.46; ratio a:c 1.25–1.30. Tooth long, sharp, strongly curved, concave ventrally with smooth surface. Intermediate sclerite 0.24–0.26 mm long, 0.15 mm wide at ventral bridge. Epipharyngeal sclerite visible only in dorsal view, with medial lobe directed anteriorly. Labial sclerite robust, sclerotized, and triangular in dorsal view. Parastomal bar extending three-fourths length of intermediate sclerite. Dorsal arch 0.33–0.35 mm high. Dorsal cornu with well–defined sclerotized area adjacent to notch, 0.64–0.74 mm long. Dorsal bridge prominently projecting anteriorly from dorsal cornu and sclerotized. Anterior sclerite irregularly shaped and sclerotized. Cornu notch (N) 0.37–0.52 mm long and cornu notch index (N/DC) 0.57–0.69. Ventral cornu with well-defined sclerotized area from notch to pharyngeal bar and grooves. Pharyngeal filter with weakly sclerotized anterior bar and eight or nine ridges forming a series of grooves along length of ventral cornu. Ventral cornu 0.93–1.01 mm long from pharyngeal bar to posterior end of grooves. Ventral cornu 1.26–1.57 × as long as sclerotized area of dorsal cornu.

***Thoracic and abdominal segments*.** Thoracic segments with dorsal spinules conical, symmetrical to slightly curved posteriorly; dorsal spinule pattern as follows: T1 with three rows; T2 with four or five rows; T3 with four rows; ventral spinule pattern as follows: T1 with 13 or 14 rows; T2 with 4–6 rows; T3 with 3–5 rows. Abdominal segments with dorsal spinules as follows: A1 with two rows; A2–A8 lacking spinules; ventral creeping welts present on all abdominal segments; ventral spinule pattern as follows: A1 with 5–8 rows; A2 with 6–9 rows; A3 with eight or nine rows; A4 with 9–12 rows; A5 with 8–12 rows; A6 with 9–11 rows; A7 with seven or eight rows; A8 with 6–9 rows. Additional three irregular rows of spinules anterior and posterior to anal lobes, lateral rows apparently absent, spinules large, conical, pointing away from anal lobes.

***Prothoracic spiracle*** (Figs 102, 103). Bilobed, bearing 22–30 tubules, distally rounded and arranged in a single, sinuous row except medially when spacing is irregular. Spiracle distal width 0.41–0.44 mm; basal width 0.18‒0.20 mm at junction with trachea.

***Caudal segment*** (Figs 104, 105). Dorsal (D1 and D2) tubercles and sensilla weakly developed; D1 distinctly anterior to D2. Intermediate tubercles I1 and I2 more strongly developed, but associated sensilla moderately developed; I1 distinctly anterior to I2. L1, V1, and V2 tubercles and associated sensilla weakly developed. Anal lobe entire and protuberant.

**Figures 106–108.**
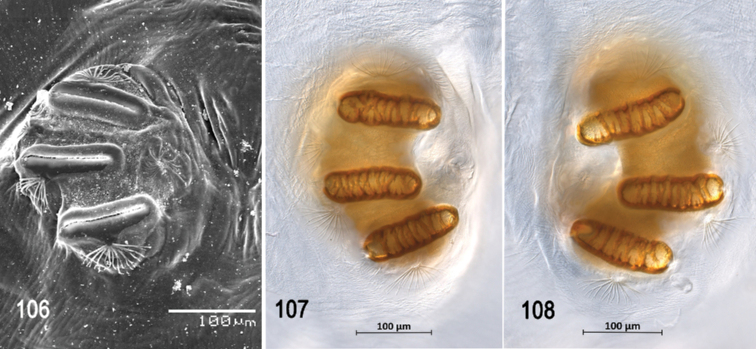
Scanning electron photomicrograph and optical photomicrographs of posterior spiracles of third instar of Anastrephasp.nr.protuberans. Scale bars: 100 μm.

***Posterior spiracle*** (Figs 104, 106–108). Located above horizontal midline. Posterior spiracle openings with thick rimae and numerous trabeculae; 122–145 µm long; 40‒48 µm wide; ratio length/width 2.8‒3.4. Ecdysial scar apparent. Felt chamber oval, 271‒305 µm in diameter at junction with trachea. Spiracular process SP-I comprising 5‒11 trunks and 9‒20 tips; ratio tips/trunks 1.4‒ 2.5; basal width 8‒11 µm; ratio basal width/length of spiracular opening 0.06‒0.08. SP-II comprising 4‒9 trunks and 11‒19 tips. SP-III comprising 4‒8 trunks and 7‒16 tips. SP-IV comprising 7‒10 trunks and 14‒21 tips; ratio tips/trunks 1.55‒2.6; basal width 9‒12 µm; ratio basal width/length of spiracular opening 0.07‒0.09.

##### Distribution.

Anastrepha sp. near protuberans is known only from Ecuador and Peru.

##### Biology.

We collected larvae of this species from fruit of *Sterculiafrondosa*, the first host plant record. The larvae feed only on the seeds of the fruit.

##### Molecular identification.

COI barcodes were generated from five larvae from Ecuador and two adults from Peru and submitted to GenBank (MT672163–MT672165, MT763909–MT763911, MT763914). The identity of the described larvae is only based on these data. K2P distances between Anastrephasp.nr.protuberans larvae and the adult sequences ranged from 0.0–1.2%. BLAST searches yielded no close matches to sequences from other *Anastrepha* species. The five larval barcodes returned consensus identifications of Anastrephasp.nr.protuberans with either three or two votes (Moore et al. in press).

#### ﻿ Anastrepha

Taxon classificationAnimaliaDipteraTephritidae

sp. Sur-16

296E5D1D-8FE9-5696-ABFB-52A1D3A8D186

Figs 109–112 , 113–118 , 119–122

##### Material examined.

Suriname • 8 larvae; Brokopondo, Bergendal Amazonia Wellness Resort; 5.1506°N, 55.0690°W; 16 m a.s.l.; 10 May 2018; A. Muller leg.; reared from fruit of *Quararibeaguianensis* Aubl. (Malvaceae); FSCA (AP20191024.03–AP20191024.07, AP20201117.01–AP20201117.03).

**Figures 109–112.**
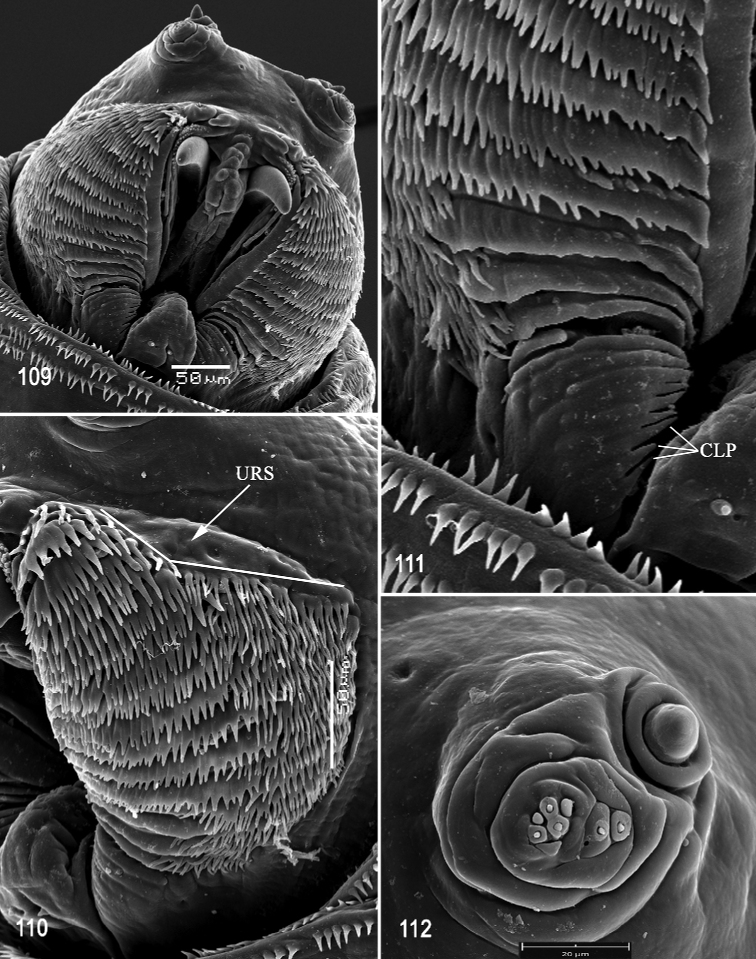
Scanning electron photomicrographs of third instar of *Anastrepha* sp. Sur-16 **109** pseudocephalon **110** oral ridges **111** comb-like processes **112** antenna and maxillary palp. Abbreviations: CLP, comb-like processes; URS, upper right section with an obtuse angle shape. Scale bars: 20 μm (**112**); 50 μm (**109, 110**).

##### Diagnosis.

The larvae of *Anastrepha* sp. Sur-16 differs from other species of *Anastrepha* in having deeply dentate posterior margin of the oral ridges and group of small cuticular processes located adjacent to the mouthhook and posterior to the preoral organ. The posterior margins of the oral ridges resemble those of *A.haplacantha*, but that species lacks the comb-like processes. It can be further distinguished from *A.haplacantha*, in having fewer oral ridges, fewer tubules on the prothoracic spiracle, and greater basal width of the posterior spiracle.

##### Description.

***Habitus*.** Third instar elongate, cylindrical, tapered anteriorly and caudal end truncate; color creamy; amphipneustic. Length 8.10‒8.60 mm and width 1.52‒1.62 mm at the sixth abdominal segment.

**Figures 113–118.**
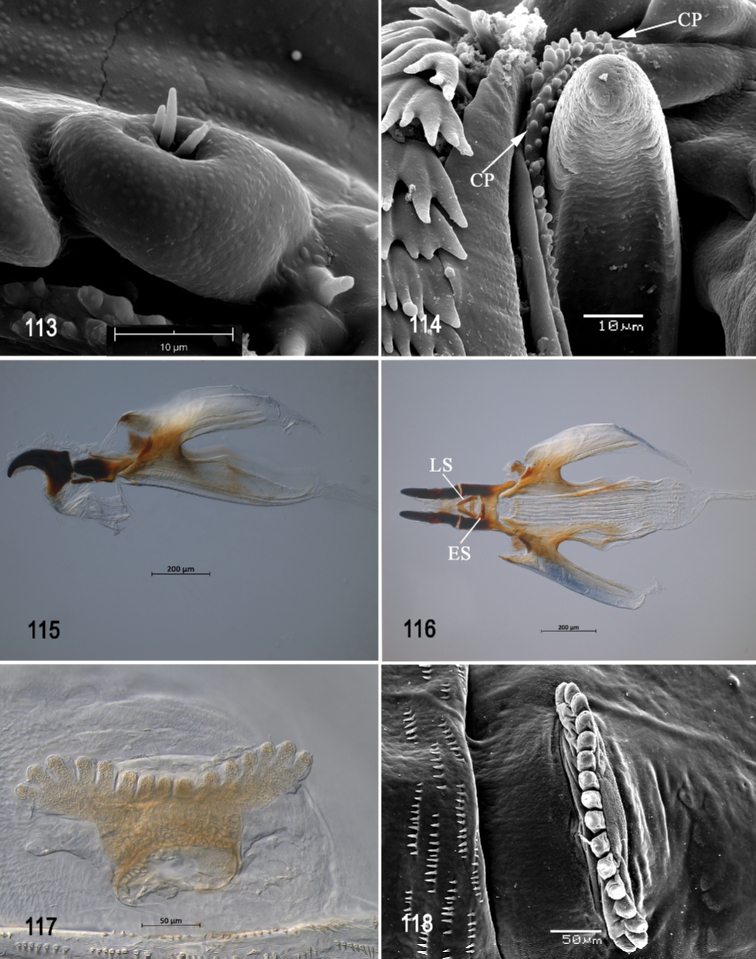
Optical photomicrographs and scanning electron photomicrographs of third instar of *Anastrepha* sp. Sur-16 **113** preoral organ **114** ventral surface of mouthhook **115** cephaloskeleton, lateral view **116** cephaloskeleton, dorsal view **117** prothoracic spiracle, lateral view **118** prothoracic spiracle, dorsolateral view. Abbreviations: CP, cuticular processes; ES, epipharyngeal sclerite; LS, labial sclerite. Scale bars: 10 μm (**113, 114**); 50 μm (**117, 118**); 200 μm (**115, 116**).

***Pseudocephalon*** (Figs 109–113). Antenna and maxillary palp on moderately developed lobe. Antenna with cylindrical base and apical knob. Maxillary palp bearing three papilla sensilla, two knob sensilla; dorsolateral group of sensilla bearing two well-developed papilla sensilla, aligned perpendicular to palp and surrounded by a collar. Facial mask partly globular in lateral view, upper right section lacking ridges and accessory plates and forming almost an obtuse angle. Preoral organ bearing 1–3 peg sensilla, located apically on a large, elongated-rounded lobe directly anterior to mouthhook; adjacent medial preoral lobe separate, short-elongate, narrow, extending partially posterior to lobe bearing preoral organ. A group of small cuticular processes arranged in at least two rows arising distally from the medial preoral lobe, located adjacent to the mouthhook and posterior to the preoral organ. Oral ridges in 13–16 rows, 10–13 anterior ridges with deeply dentate margins, projections closely spaced, two or three posterior ridges with entire margins; numerous accessory plates present covering a much smaller area than oral ridges, with fringed posterior margins, medial and posterior plates in two or more series; 7–9 comb-like processes adjacent to labium. Labium triangular, anterior surface knobby, ventrally with two visible sensilla.

***Cephaloskeleton*** (Figs 114–116). Total length from tip of mouthhook to end of ventral cornu 1.13–1.18 mm. Mouthhook well sclerotized, black apically and basally; length a 0.22–0.23 mm; length b 0.16–0.17 mm; height c 0.16–0.17 mm; ratio a:b 1.30–1.41; ratio a:c 1.34–1.40. Tooth long, sharp, strongly curved, concave ventrally with eroded surface. Intermediate sclerite 0.20–0.21 mm long, 0.13–0.14 mm wide at ventral bridge. Epipharyngeal sclerite visible only in dorsal view, with medial lobe directed anteriorly. Labial sclerite robust, sclerotized, and triangular in dorsal view. Parastomal bar extending three-fourths length of intermediate sclerite. Dorsal arch 0.25–0.26 mm high. Dorsal cornu with well-defined sclerotized area adjacent to notch, 0.50–0.54 mm long. Dorsal bridge prominently projecting anteriorly from dorsal cornu and slightly sclerotized. Anterior sclerite irregularly shaped and sclerotized. Cornu notch (N) 0.30–0.35 mm and cornu notch index (N/DC) 0.61–0.66. Ventral cornu with well-defined sclerotized area from notch to pharyngeal bar and grooves. Pharyngeal filter with weakly sclerotized anterior bar and eight ridges forming a series of grooves along length of ventral cornu. Ventral cornu 0.73–0.73 mm long from pharyngeal bar to posterior end of grooves. Ventral cornu 1.40–1.49 × as long as sclerotized area of dorsal cornu.

***Thoracic and abdominal segments*.** Thoracic segments with dorsal spinules conical, symmetrical to slightly curved posteriorly; dorsal spinule pattern as follows: T1 with five rows, forming scalloped plates; T2 with three rows; T3 lacking spinules; ventral spinule pattern as follows: T1 with ten rows; T2 with three or four rows; T3 with one or two rows. Abdominal segments all lacking dorsal spinules; ventral creeping welts present on all abdominal segments; ventral spinule pattern as follows: A1 with three rows, A2 with six or seven rows; A3 with 6–10 rows, A4 with eight or nine rows; A5 to A7 with seven or eight rows; A8 with 6–9 rows. Additional three irregular rows of spinules anteriorly and posteriorly to anal lobes, one or two rows laterally, spinules large, conical, pointing away from anal lobes.

**Figures 119–122.**
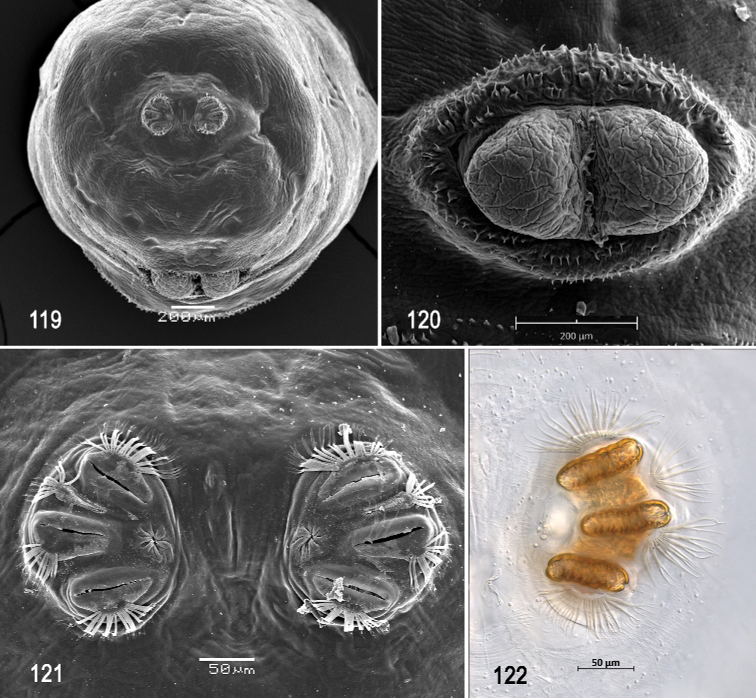
Scanning electron photomicrographs and optical photomicrograph of third instar of *Anastrepha* sp. Sur-16 **119** caudal segment **120** anal lobe **121, 122** posterior spiracle. Scale bars: 50 μm (**121, 122**); 200 μm (**119, 120**).

***Prothoracic spiracle*** (Figs 117, 118). Bilobed, bearing 12–17 tubules, distally rounded and arranged in a single sinuous row. Spiracle distal width 0.23–0.28 mm; basal width 0.09‒0.11 mm at junction with trachea.

***Caudal segment*** (Figs 119, 120). Dorsal (D1) tubercles moderately developed, D2 tubercles and associated sensilla weakly developed; D1 distinctly anterior to D2. Intermediate tubercles I1 and I2 more strongly developed, but associated sensilla moderately developed; I1 distinctly ventral to I2. L1, V1 and V2 tubercles and associated sensilla weakly developed. Anal lobe entire and protuberant.

***Posterior spiracle*** (Figs 119, 121, 122). Located above horizontal midline. Posterior spiracle openings with thick rimae and numerous trabeculae; 69–80 µm long; 24‒27 µm wide; ratio length/width 2.9‒3.0. Ecdysial scar apparent. Felt chamber oval, 129‒168 µm in diameter at junction with trachea. Spiracular process SP-I comprising 13‒18 trunks and 19‒34 tips; ratio tips/trunks 1.5‒1.8; basal width 29‒36 µm; ratio basal width/length of spiracular opening 0.39‒0.44. SP-II comprising 5‒8 trunks and 7‒18 tips. SP-III comprising 8‒13 trunks and 14‒24 tips. SP-IV comprising 13‒17 trunks and 25‒40 tips; ratio tips/trunks 1.92‒2.35; basal width 23‒34 µm; ratio basal width/length of spiracular opening 0.33‒0.45.

##### Distribution.

*Anastrepha* sp. Sur-16 is known only from Suriname (Brokopondo).

##### Biology.

We reared this species from fruit of *Quararibeaguianensis*, the first host plant record. Larvae feed on the pulp.

##### Molecular identification.

COI barcodes were generated from five larvae and two adults and submitted to GenBank (MT644074–MT644078, MT672219–MT672220). These data further confirm the identity of the described larvae. K2P distances between *Anastrepha* sp. Sur-16 larvae and the adult sequences ranged from 0.02–1.2%. BLAST searches yielded no close matches to sequences of other *Anastrepha* species. The five larval barcodes returned consensus identifications of *Anastrepha* sp. Sur-16 with either three or two votes (Moore et al. in press).

## ﻿Discussion

The extraordinary morphology of the pseudocephalon of third instars of the species of the *mucronota* group treated in this study includes characters that appear to be relevant to analysis of the phylogenetic relationships of this species group. Norrbom et al. (1999) recognized the *mucronota* group for 31 species but indicated that it could be paraphyletic. It included species without a strong crease in the proctiger (a plesiomorphic state) but lacking synapomorphies of other species groups with this character state. Two wing characters common within the group (C- and S-bands separated; vein R_2+3_ sinuous) were mentioned as possibly of phylogenetic significance, but they are not consistent nor unique to the group. Additional species have subsequently been described or transferred to the *mucronota* group such that it currently includes 54 described and a number of as yet undescribed species (Norrbom et al. 2012; Moore et al. in press). Mengual et al. (2017) included 19 described and four undescribed species (sp. 4 and sp. nr. submunda are now believed to be the same species) that are currently placed in the *mucronota* group. In their maximum likelihood tree (Fig. 3), these species were placed in four clades comprising, respectively, 14 species, four species (with species of the *raveni* group), two clades with two species each, and one species grouped with a species of the *schausi* group. The *mucronota* group thus may indeed not be monophyletic, but support for some of the intermediate branches was low enough that the relationships of some species remain unclear.

**Figure 123.**
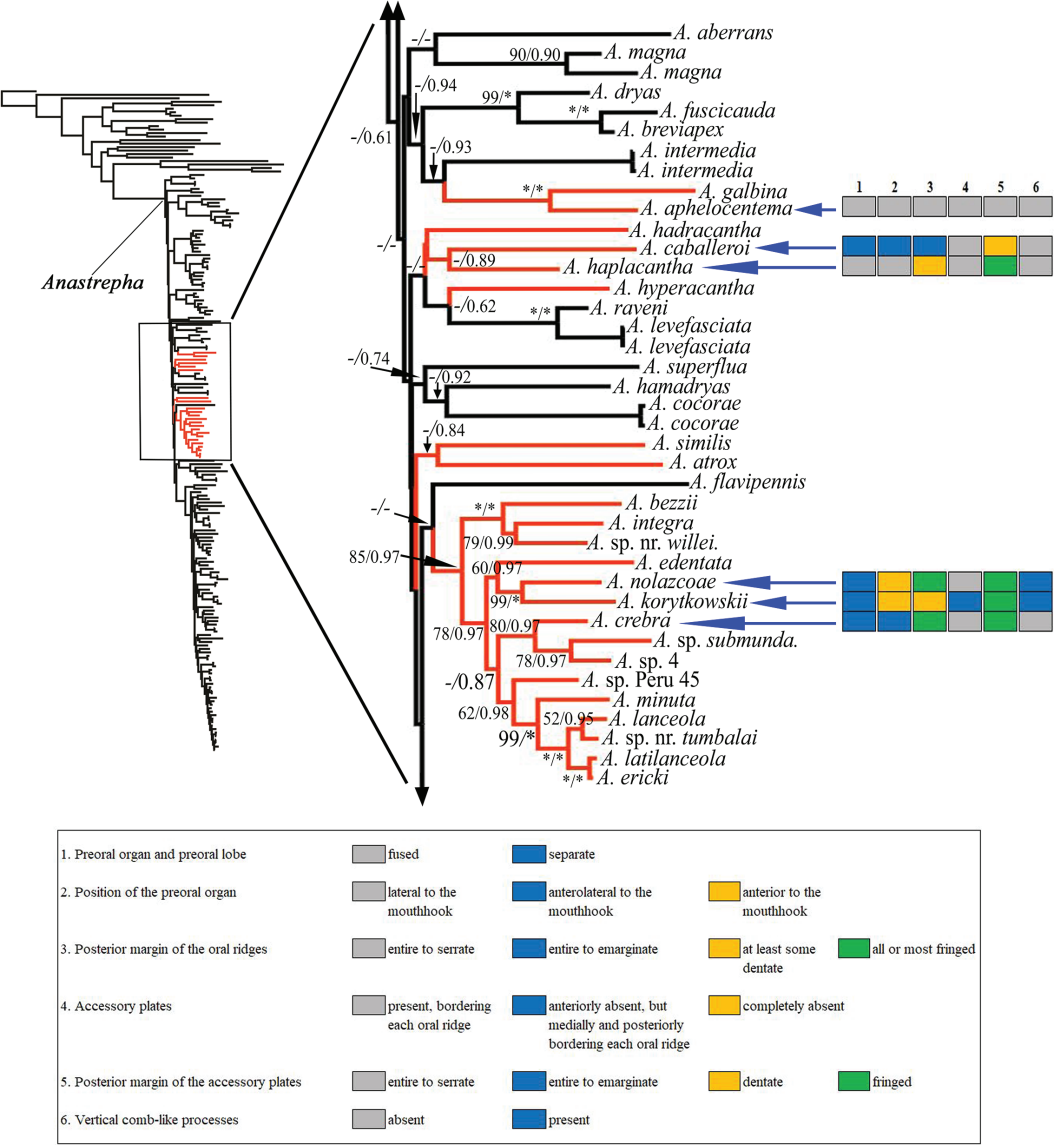
Tree visualization of the novel characters of the pseudocephalon. *Anastrepha* phylogeny and relationships of species within the *mucronota* group (clades and branches in orange) were taken from Mengual et al. (2017). The larval characters are indicated for the six selected traits: (1) Preoral organ and preoral lobe; (2) Position of the preoral organ; (3) Posterior margin of the oral ridges; (4) Accessory plates; (5) Posterior margin of the accessory plates; (6) Vertical comb-like processes.

Of the nine species for which larvae are described in this paper, six were included by Mengual et al. (2017): *A.aphelocentema* was clustered with *A.galbina* Stone rather distant from the other species; *A.caballeroi* and *A.haplacantha* were placed in the clade with the species of the *raveni* group; and *A.crebra*, *A.korytkowskii*, and *A.nolazcoae* were in the largest cluster of 14 species. Because the number of species for which larvae are known is still very limited and the number of larval morphological characters that appear useful for phylogenetic analysis is also small, we consider it premature to undertake a rigorous analysis at this time. However, to explore the potential of the novel larval characters for this purpose, we plotted these characters onto the Mengual et al. (2017: fig. 1, partially redrawn here as Fig. 123) tree. We discuss each character, indicate the species in which the apomorphic states occur and where these species are placed on the tree, and speculate regarding the significance of these character states.

The characters with new character states are the size and shape of the preoral lobe bearing the preoral organ, the position of the preoral organ, and the posterior margins of the oral ridges and accessory plates (see Tables 5, 6). The position of the preoral organ anterior to the mouthhook in *A.korytkowskii*, *A.nolazcoae*, *Anastrepha* sp. Peru-82, and *Anastrepha* sp. Sur-16, and anterolateral to the mouthhook in *A.caballeroi* and *A.crebra* are unique character states within *Anastrepha* and found only in these six species and *A.curvicauda* (anterior to mouthhook) (Figs 18, 31, 53, 66, 81, 109; character 2). The separation of the preoral organ from the preoral lobe is only found in the six species of the *mucronota* group above, *A.grandis*, *A.leptozona*, *and A.pickeli* (Table 6), although in *A.grandis*, *A.leptozona*, and *A.pickeli* the preoral organ is lateral to the mouthhook (see Frías et al. 2009: fig. 4 and Dutra et al. 2018a: fig. 2A). In most *Anastrepha* species, the preoral organ is lateral to the mouthhook, on the end of a long simple preoral lobe, not on a separate cylindrical lobe (e.g., as in *A.aphelocentema*, *A.haplacantha*, and Anastrephasp.nr.protuberans (Figs 1, 41, 44, 95). The position of the preoral organ anterior to the mouthhook has not been observed in other genera of Tephritidae, however, the presence of a separate cylindrical lobe bearing the preoral organ does occur in many genera of Dacinae, such as *Bactrocera*, *Dacus*, and *Zeugodacus* of the Dacini, *Ceratitis* of the Ceratitidini, and *Acroceratitis* and *Ichneumonopsis* of the Gastrozonini (White and Elson-Harris 1992; Carroll et al. 2004; Kovac et al. 2013; Schneider et al. 2018). The position of the preoral organ anterolateral or anterior to the mouthhook (apomorphic states) in most species of the *mucronota* group that we examined suggests that this character has phylogenetic signal, although the plesiomorphic state in *A.haplacantha* and A.sp.nr.protuberans, as well as *A.aphelocentema*, suggests that these species may be less closely related than the other species or that there is some homoplasy in this character.

**Table 5.** Larval characters and character states used for comparative morphology of the pseudocephalon.

Character	State
1. Preoral organ and preoral lobe	0, fused; 1, separate
2. Position of the preoral organ	0, lateral to the mouthhook; 1, anterolateral to the mouthhook; 2, anterior to the mouthhook
3. Posterior margin of the oral ridges	0, entire to serrate; 1, entire to emarginate; 2, at least some dentate; 3, all or most fringed
4. Accessory plates	0, present, bordering each oral ridge; 1, anteriorly absent, but medially and posteriorly bordering each oral ridge; 2, completely absent
5. Posterior margin of the accessory plates	0, entire to serrate; 1, entire to emarginate; 2, dentate; 3, fringed
6. Vertical comb-like processes	0, absent; 1, present

**Table 6.** Character matrix of the outgroup and ingroup taxa used for comparative morphology of the pseudocephalon.

Species group	Species	Characters					
		1	2	3	4	5	6
	Outgroup						
*curvicauda*	*A.curvicauda*	0	2	0	0	0	0
*fraterculus*	*A.amita*	0	0	1	0	?	0
	*A.amplidentata*	0	0	1	0	0	0
	*A.bahiensis*	0	0	1	0	1	0
	*A.coronilli*	0	0	1	2	–	0
	*A.durantae*	0	0	1	0	1	0
	*A.ludens*	0	0	1	0	1	0
	*A.sororcula*	0	0	1	0	1	0
	*A.suspensa*	0	0	1	0	1	0
	*A.zenildae*	0	0	1	0	1	0
*grandis*	*A.grandis*	1	0	1	0	1	0
*leptozona*	*A.leptozona*	1	0	0	0	0	0
*pseudoparalella*	*A.limae*	0	0	1	0	1	0
*serpentina*	*A.pulchra*	0	0	1	0	1	0
	*A.serpentina*	0	0	1	0	1	0
*spatulata*	*A.pickeli*	1	0	1	0	1	0
*striata*	*A.striata*	0	0	1	0	1	0
	Ingroup						
*mucronota*	*A.aphelocentema*	0	0	0	0	0	0
	*A.caballeroi*	1	1	1	0	2	0
	*A.crebra*	1	1	3	0	3	0
	*A.haplacantha*	0	0	2	0	3	0
	*A.korytkowskii*	1	2	2	1	3	1
	*A.nolazcoae*	1	2	3	0	3	1
	*Anastrepha* sp. Peru-82	1	2	3	1	3	0
	Anastrephasp.nr.protuberans	0	0	3	0	3	0
	*Anastrepha* sp. Sur-16	1	2	2	0	3	1

(?) Unknown data from previous studies. (–) Inapplicable data because the accessory plates are absent in *A.coronilli*.

In all previously described larvae of *Anastrepha*, the posterior margins of the oral ridges (character 3) and accessory plates are variously entire, serrate, occasionally incised, sparsely emarginate, or scalloped; in none of these species are the margins dentate or fringed (Steck et al. 1990; Carroll et al. 2004; Dutra et al. 2018b; Rodriguez et al. 2021). Although there is some variation in this character in other, rather distantly related genera (e.g., *Rioxoptilonadunlopi* (Wulp), *Rioxoptilonaochropleura* (Hering), and *Rioxoptilonavaga* (Wiedemann) of the subfamily Phytalmiinae, tribe Acanthonevrini, *Bactrocerabryoniae* (Tryon), *Bactroceracarambolae* Dew and Hancock, *Bactrocerafrauenfeldi* (Schiner), *Bactrocerajarvisi* (Tryon), *Bactroceralatifrons* (Hendel), *Bactroceramusae* (Tryon) of the subfamily Dacinae, tribe Dacini, *Anoplomusrufipes* Hardy, *Chaetellipsisalternata* (Zia), *Chaetellipsis* sp., *Cyrtostolalimbata* (Hendel), and *Paraxarnuraanephelobasis* Hardy of the subfamily Dacinae, tribe Gastrozonini) (see Elson-Harris 1992: pls 3, 8, 14, 93, 314; White and Elson-Harris 1992: pls 9a, 14a, 17b, 18c, 20b: Schneider et al. 2017: figs 1E, 4E, 7D, 7E, 12D, 12E), the dentate and fringed margins of the accessory plates appear to be apomorphic character states (see Table 6), and one or both states are present in species of the *mucronota* group, except for *A.aphelocentema*, in which they are finely serrate or entire. That and the dentate or fringed (i.e., more deeply incised) margins of the oral ridges could be synapomorphies for a large portion of the species within the *mucronota* group (with some homoplasy involving states 2 and 3 of these characters) or alternatively could have arisen independently in the clade including *A.crebra*, *A.korytkowskii*, and *A.nolazcoae* and that containing *A.caballeroi* and *A.haplacantha* (perhaps with reversal in character 3 in *A.caballeroi*) if the relationships among these species in the tree of Mengual et al. (2017) are correct (Fig. 123). *Anastrepha* sp. Peru-82, A.sp.nr.protuberans, and *A.* sp. Sur-16 also share these apomorphic character states (Table 6), supporting their inclusion in the *mucronota* group. The accessory plates (Figs 14, 15) covering a much larger area than the oral ridges appear to be an autapomorphy of *A.caballeroi*.

Another remarkable feature reported for the first time in *Anastrepha* is the vertical comb-like processes on the margin of the oral cavity found only in *A.korytkowskii*, *A.nolazcoae*, and *Anastrepha* sp. Sur-16 (Figs 53, 68, 111). These processes are exceptional and absent in other tephritid larvae described to date. This morphological feature appears to be a synapomorphy of *A.korytkowskii* and *A.nolazcoae*, which are sister taxa in the Mengual et al. (2017) tree (Fig. 123, Table 6), and perhaps some other closely related taxa (but not *A.crebra*). We hypothesize that *Anastrepha* sp. Sur-16, which also has the apomorphic state, is also very closely related to these two species based on this character and those previously discussed (fringed margins of the accessory plates and preoral organ located anterior to mouthhook).

Our results support the hypothesis of Mengual et al. (2017) that *A.aphelocentema* is an outlier from the *mucronota* group, as it possesses no apomorphic larval character states. In particular, the posterior margins of the oral ridges and accessory plates are entire to finely serrate (plesiomorphy) (Figs 2, 123, Table 6), whereas in the other species the margins of the accessory plates are dentate (*A.caballeroi*, Figs 15, 123, Table 6) or fringed (the other four species, *A.crebra*, *A.haplacantha*, *A.korytkowskii*, *A.nolazcoae*, Figs 29, 42, 54, 67, 123, Table 6). The fringed margins of the accessory plates, and the at least sometimes dentate and fringed margins of the oral ridges of those other four species are distributed across the *mucronota* group in two distant clades (Fig. 123). Within the well-supported clade with 14 taxa, the location of the preoral organ (anterior to mouthhook) and the preoral lobe and preoral organ (separate) supports the relationship of *A.korytkowskii* + *A.nolazcoae* and may indicate that *Anastrepha* sp. Peru-82 + *Anastrepha* sp. Sur-16 belong to this clade (Table 6). Of these four species, *A.korytkowskii*, *A.nolazcoae*, and *Anastrepha* sp. Sur-16 appear to be more closely related due to the presence of the vertical comb-like processes (Table 6). The comparative morphology of the pseudocephalon concurs with the molecular phylogeny of Mengual et al. (2017), with the exception of *A.caballeroi* and *A.haplacantha* not sharing derived larval morphological character states (although presence of states 5.2 or 5.3, i.e., more incised accessory plates, could be considered a synapomorphy if these states are interpreted as part of a transformation series). It also suggests that *Anastrepha* sp. Peru-82, Anastrephasp.nr.protuberans, and *Anastrepha* sp. Sur-16 may belong to the largest and well-supported clade (Table 6, Fig. 123). However, the relationships of these and all of the species of the *mucronota* group should be further evaluated and confirmed from additional molecular and morphological phylogenetic analysis as samples of larvae and adults become available.

## Supplementary Material

XML Treatment for Anastrepha aphelocentema

XML Treatment for Anastrepha caballeroi

XML Treatment for Anastrepha crebra

XML Treatment for Anastrepha haplacantha

XML Treatment for Anastrepha korytkowskii

XML Treatment for Anastrepha nolazcoae

XML Treatment for Anastrepha

XML Treatment for Anastrepha sp. near protuberans

XML Treatment for Anastrepha
